# Modelling with ANIMO: between fuzzy logic and differential equations

**DOI:** 10.1186/s12918-016-0286-z

**Published:** 2016-07-27

**Authors:** Stefano Schivo, Jetse Scholma, Paul E. van der Vet, Marcel Karperien, Janine N. Post, Jaco van de Pol, Rom Langerak

**Affiliations:** 1 Formal Methods and Tools, Faculty of EEMCS, University of Twente, P.O. Box 217, Enschede, 7500AE The Netherlands; 2 Developmental BioEngineering, MIRA Institute for Biomedical Technology and Technical Medicine, University of Twente, P.O. Box 217, Enschede, 7500AE The Netherlands; 3 Human Media Interaction, Faculty of EEMCS, University of Twente, P.O. Box 217, Enschede, 7500AE The Netherlands

**Keywords:** Modelling, Signalling pathway, Timed automata, Dynamic behaviour

## Abstract

**Background:**

Computational support is essential in order to reason on the dynamics of biological systems. We have developed the software tool ANIMO (Analysis of Networks with Interactive MOdeling) to provide such computational support and allow insight into the complex networks of signaling events occurring in living cells. ANIMO makes use of timed automata as an underlying model, thereby enabling analysis techniques from computer science like model checking. Biology experts are able to use ANIMO via a user interface specifically tailored for biological applications. In this paper we compare the use of ANIMO with some established formalisms on two case studies.

**Results:**

ANIMO is a powerful and user-friendly tool that can compete with existing continuous and discrete paradigms. We show this by presenting ANIMO models for two case studies: *Drosophila melanogaster* circadian clock, and signal transduction events downstream of TNF *α* and EGF in HT-29 human colon carcinoma cells. The models were originally developed with ODEs and fuzzy logic, respectively.

**Conclusions:**

Two biological case studies that have been modeled with respectively ODE and fuzzy logic models can be conveniently modeled using ANIMO. The ANIMO models require less parameters than ODEs and are more precise than fuzzy logic. For this reason we position the modelling paradigm of ANIMO between ODEs and fuzzy logic.

**Electronic supplementary material:**

The online version of this article (doi:10.1186/s12918-016-0286-z) contains supplementary material, which is available to authorized users.

## Background

### Modelling in cell biology

Executable biology [1] is a young subfield in computational modelling, aimed at constructing models that mimic biological phenomena in silico. It provides an interesting paradigm to enhance network diagrams with an underlying formal description of network components and their interactions. For this purpose a wealth of different modeling paradigms has been proposed (see [2] for an overview). Several approaches consist in the abstraction of continuous models into discrete transition systems (e.g. [3–6]); this may enable the use of model checking as a state space exploration technique [3, 7, 8]. Our approach is based on Timed Automata models [9] defined by linear approximations (with arbitrary precision) of ordinary differential equations (ODEs); this has the benefit of using existing mature Timed Automata analysis techniques. It is not the ambition of this paper to exhaustively compare this approach with all existing formalisms; instead, we want to show that this model has resulted in an effective and user-friendly tool, which compares favorably to some prominent approaches, most notably ODEs and fuzzy logic. We have developed ANIMO (Analysis of Networks with Interactive MOdelling, [10, 11]), a software tool that provides an enabling technology to increase the use of computational models by experimental biologists using their domain-specific language, i.e. the representation of a biochemical network as a graph where each node identifies a molecular species and each edge an interaction. ANIMO enriches the normally static biological network diagrams with dynamic information, which is then used to automatically produce formal models representing the biological network. Such models are indispensable for formally comparing experimental data with prior knowledge, or for structuring experimental findings into a new theory. When dealing with complex biological networks, executable biology models are particularly useful to understand the non-linear dynamics and the entailed emergent properties of the networks. In those cases, an ANIMO model can be used as a support to obtain insight based on abstract representations of the interactions occurring inside living cells. Other applications of ANIMO models include performing in silico experiments and the storage and transfer of knowledge on biological networks.

### An introduction to ANIMO

**Fig. 1:**
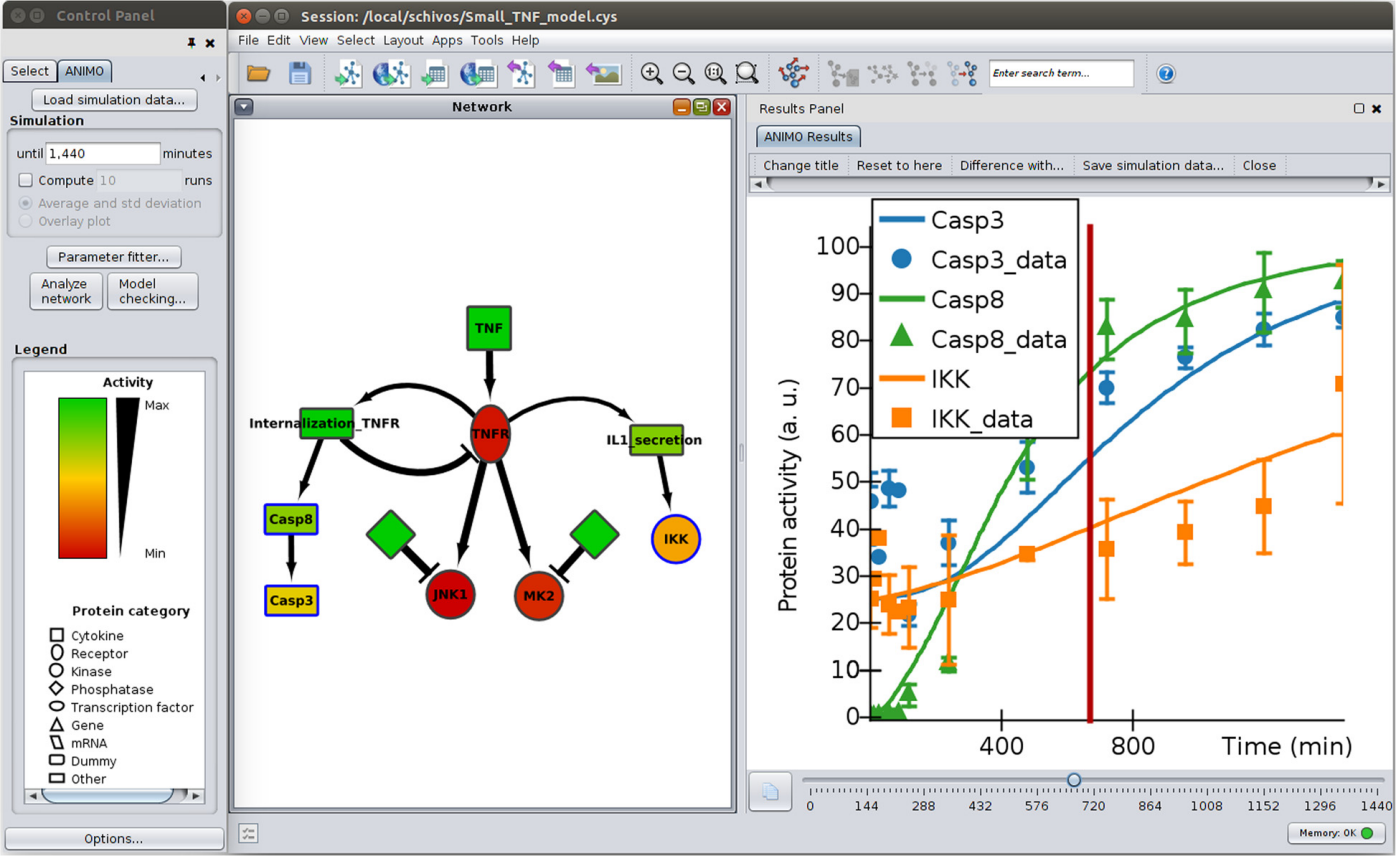
The Cytoscape user interface running the ANIMO plug-in. The *Network* panel in the centre contains the nodes-edges model of the example TNF *α* pathway (see Methods section), with colours indicating node activity levels and shapes representing different protein categories (see the *Legend* on the left). The *Results Panel* on the right contains a graph plotting activity levels of selected nodes during the first 24 hours of simulation of the model. The slider under the graph allows the user to select the time instant (marked as a vertical red line in the graph) on which the colours of the nodes in the *Network* are based. The series with the _data suffix is experimental data from [26], considering a treatment with 100 ng/ml TNF *α*. All acronyms used in this paper and their corresponding UniProt IDs are listed in Additional file 1: Section C

The user interface of ANIMO is displayed in Fig. 1, where we present an example of a biological network enriched with dynamic information. ANIMO is implemented as a plug-in to Cytoscape [12] (both the old 2.8.x and the new 3.x versions are supported), a software tool developed to represent biological networks. On top of the static topological information displayed in Cytoscape, ANIMO represents biological interactions starting from the basic concept of *activity*: each biological entity in an ANIMO model is considered to be either active or inactive. Activity is to be interpreted in a very broad sense: for example, an active gene is being transcribed, an active kinase can perform phosphorylations, etc. Each node in an ANIMO network represents both active and inactive entities of the same type, with the relative amount of active entities (the *activity level*) represented by the node colour on a user-configurable scale. Interactions among nodes define how the biological entities in a network influence each other’s activity. Only nodes whose activity level is larger than 0 (such nodes are called *active*) can have an influence on their downstream targets, and only if that influence is not counterbalanced by intervening opposite interactions. For example, the interaction *A*→*B* (read “A activates B”) indicates that node A, if active, will increase the activity level of node B. If we add an additional interaction to the example, *C*⊣*B* (“C inhibits B”), with C also active, then the activity level of B will change depending on the activity levels of A and C, and on their quantitative influence. The influence of an ANIMO interaction is quantified by a parameter *k*, which defines the speed at which that interaction occurs: higher values of *k* give faster interactions. These *k*-values are the only parameters needed in an ANIMO model, and can be given in either a quantitative (as real numbers) or a qualitative way, choosing among self-explanatory descriptions such as “very slow”, “slow”, “medium”, “fast”, “very fast”.

ANIMO was conceived to model signalling pathways, and in this context it is sensible to assume that the concentration of the involved molecular species does not change noticeably in the considered time span (several minutes to a few hours). For this reason, ANIMO models use the activity level of a node to represent the fraction of molecules that are “active”, while the total amount of molecules is assumed to remain constant. However, this assumption is not as restrictive as it seems: ANIMO models can still represent protein concentration by identifying it with activity. For example, we can represent production of protein *A* with a process increasing the activity level of node *A*, while degradation will decrease it.

The concise language used in ANIMO to represent reaction information is powerful enough to model various types of interactions. For example, we can easily translate Boolean OR gates: (A OR B) → C becomes the two separate reactions A → C and B → C. This means that whenever either A or B is active, C will eventually be activated, i.e. reaction effects are always additive. This representation of OR is thus non-exclusive, so C will be activated also if both A and B are active, but in that case the activation will proceed faster. A Boolean AND gate can be explicitly represented with the “AND” approximation scenario (see Additional file 1: Section A.1): with (A AND B) → C, C will be activated only if both A and B are active.

Combining these basic tools makes the representation of more complex Boolean formulas also possible, by properly combining the basic gates. The same is generally true also if we move from Boolean interactions to precise kinetic formulas. We note that in this case some specific mathematical functions (square root, exponential, …) may be needed. It is in principle possible to correctly translate all those functions into the underlying Timed Automata model, even if it may require some effort. However, as such functions are normally used to represent a complex mechanism in abstract form, we advise to use ANIMO with the same aim, i.e. as a tool to abstractly represent complex mechanisms. As an example, see the representation of the day/night cycle with a repressilator construct [13] in ANIMO as opposed to the piecewise linear approximation used in the model we use as reference (see Results and Additional file 1: Section B.1).

ANIMO produces graphs showing how the activity levels of selected nodes change over time, allowing the user to obtain a view on the dynamic behaviour of their network. In order to obtain these results, a model defined in ANIMO is automatically translated into its corresponding representation as a network of Timed Automata [9] and then analysed *behind the scenes* with the software tool UPPAAL [14]. The formal language of Timed Automata allows to represent and analyse complex behaviours precisely and efficiently, but the user does not need to directly manipulate Timed Automata or UPPAAL, as the analysis process is made transparent. A curious user can still access the underlying models and perform other analyses in UPPAAL, but that is not required in order to fully profit from ANIMO.

A detailed description on how the Timed Automata models defined by ANIMO work, and how the results are obtained, can be found in [10]. The choice of parameters for ANIMO models is described in [15] and summarized in Additional file 1: Section A.5. Additional guidance on the design of ANIMO models and how to best profit from biological experimental data can be found in [11]. The ANIMO web page [16] contains a link to the user manual and instructions to install ANIMO on a computer.

**Fig. 2:**
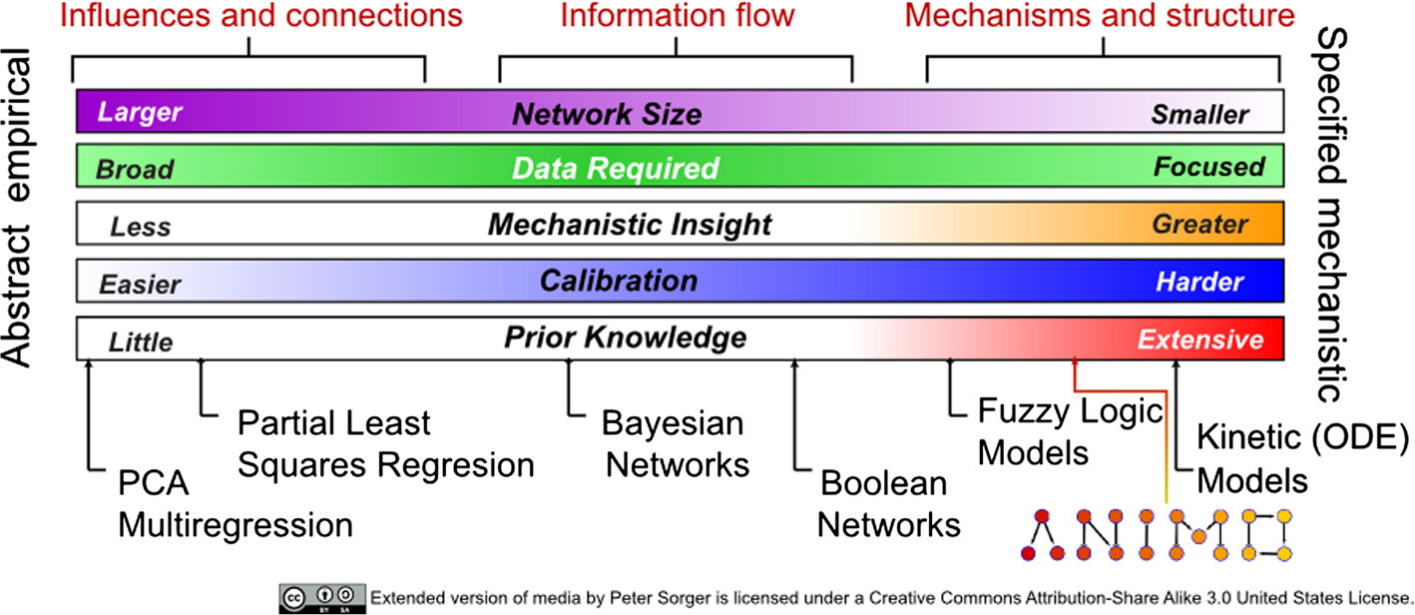
The spectrum of modelling methods, with the addition of ANIMO. The precision of ANIMO models is halfway between fuzzy logic and ODE. Compared to other modelling tools, ANIMO allows for an easier modelling experience thanks to a user-friendly interface based on the widely used network modelling software Cytoscape

Figure 2 shows the position of ANIMO in a spectrum of modelling methods. Boolean and Fuzzy Logic are based mainly on discrete transitions, whereas ordinary differential equations (ODEs) form a purely continuous model. ANIMO takes a position in between: it is based on piecewise linear approximations [17, 18] (with arbitrary precision) of ODE models. On the one hand, this means that the precision of a model can easily be tailored towards the precision and availability of the biological data. And on the other hand, this means that a model results in a (discrete) finite state space. This is important, as it enables the application of model checking techniques, which allow us to automatically explore all the possible behaviours of a model. Such techniques can be used to explore possible therapeutic avenues: by introducing some degree of freedom in the dosage of the “inputs” to the network and applying model checking, we can look for a suitably useful network state. For example, if the initial state of a network represents a cell in an *ill* state, all possible dosages of different drugs can be automatically explored through model checking, looking for a way to reach a state in which the cell is not *ill* anymore. The trace resulting from the model checking can then be used as guidance for further model refinement and investigation in the laboratory.

In the rest of the paper, we will show two case studies where models built with ANIMO are compared to models built with ODEs and Fuzzy logic. We will then compare ANIMO with other tools, focusing on the user experience of the modeller: as highlighted before, we believe that providing a suitable access to modelling formalisms is essential for their widespread application in biological and biomedical research.

## Results

### Modelling oscillation in Drosophila Melanogaster circadian clock

To demonstrate that results obtained with ANIMO are comparable to results from widely used modelling approaches, we present an ANIMO model of the circadian clock in *Drosophila Melanogaster* (they are usually in the form “model available in Additional file 2”). This ANIMO model is based on [19], where ordinary differential equations (ODEs) were used. The cyclic behaviour of the circadian clock is based on the alternating formation and destruction of the CYC/CLK protein complex. Concentration levels of this complex are in turn regulated by a series of proteins which are produced as a consequence of CYC/CLK formation. The CWO protein is central to the functioning of the network, as it degrades the mRNA for most of the involved proteins. As such, CWO acts as an inhibitor that counterbalances the effect of CYC/CLK. The positive influence of the light-regulated cryptochrome CRY on the degradation of TIM is a consequence of the passage between day and night, allowing the circadian clock to synchronize to a time zone.

**Fig. 3:**
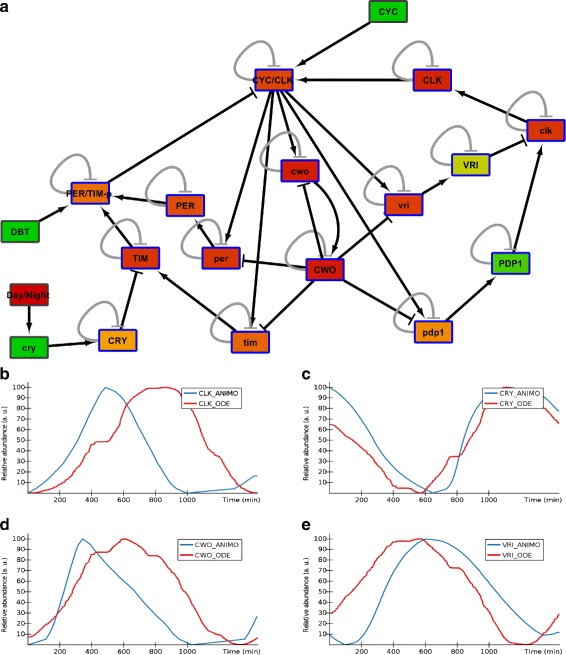
ANIMO model of the circadian clock in *Drosophila Melanogaster*. **a** The topology of the ANIMO model is the same as the model in [19]. Negative self-feedback loops are present on each of the nodes of the network to ensure that protein levels decrease over time when activating inputs are absent. This describes the normally occurring degradation of proteins in a similar way to what is done in the original ODE model. The feedback loops are represented in a lighter gray colour to enhance readability. **b**–**e** Comparing the result of the ANIMO model of *Drosophila Melanogaster* circadian clock with the model of [19]. 24 hours simulations of the two models were compared against each other, synchronizing their start point as much as possible. The blue line is the ANIMO model (_ANIMO series), while the red line represents the data computed from the original ODE model (_ODE series) using Matlab®;. The activity levels of the _ANIMO series were manually rescaled on a [0, 100] interval, to reflect what is done in the ODE model and thus facilitate comparison. Naming conventions follow the same rules as in [19], with lower-case names representing mRNA, and upper-case names indicating proteins

The ANIMO model we present here was built using the network topology presented in [19] (cf. Fig. 3a with Fig. 1 from [19]) and the same parameter settings. In order to make the amplitude of some oscillations more visible, the parameters were adapted using the techniques available in ANIMO [15].

The output of the final ANIMO model (Fig. 3a) matches the original ODE model. In particular, starting from the same initial conditions, both models achieve an oscillatory behaviour with similar periods and phases: see Fig. 3b–e for some examples, and Additional file 1: Figure S6 for the complete comparison.

**Fig. 4:**
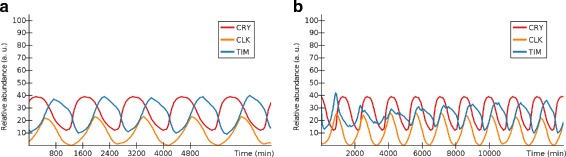
Experiment: circadian clock resynchronization in ANIMO. **a** The oscillations in CRY, CLK and TIM in normal conditions over a time span of 5 days (1 day = 1 oscillation period). **b** Recovery of circadian clock synchronization. After having altered the light/dark alternance by changing CRY’s phase, a simulation of 10 days has been performed. We note that the changes in TIM caused by CRY make the peaks in CLK gradually realign with CRY, effectively causing the resynchronization of the circadian clock to the new time zone. Similarly to what was reported in [19], the synchronization is almost complete after about 5 days. The activity levels of the series shown here were not rescaled on a [0, 100] interval as was done in the other figures for this model. This allows us to both make the series more easily distinguishable and to show how the resulting simulation would appear to an ANIMO user after following the steps we described here

A number of the experiments proposed in the original paper were also tried in the ANIMO model and gave comparable results. In particular, we note that after artificially changing the light/dark cycle, the circadian clock correctly resynchronizes to the new environmental situation. Another experiment involved the knock-out of essential nodes in the network (CLK, CYC, DBT): removing any such node removes also the oscillatory behavior, making the model reach an equilibrium point shortly after the modification. Finally, we also noted that altering the effectiveness (i.e. changing the *k* values downstream) of critical nodes such as CLK/CYC changes the period of the oscillations. All these experiments can be done directly in ANIMO’s user interface, and require few mouse clicks each. For example, the knock-out of a node can be done in ANIMO by *disabling* that node: in this way, the node will not be taken into account in any ensuing simulation. As a representative of the experiments, we show here the procedure we used to test the effects of changing the light/dark cycle. It can be noted that the procedure is more involved than disabling a node from its pop-up menu, yet it follows a consequential reasoning:

- perform an initial simulation of e.g. 24 h;
- using the slider under the computed graph, select a point where CRY is low, which corresponds to night time;
- pressing the *copy* button next to the slider, to set the currently selected point in time to be the initial configuration for all further simulations;
- disable most of the network, keeping enabled only the part of the network that changes CRY’s activity level;
- let CRY advance on itself (i.e., the light/dark cycle desynchronizes with the internal circadian clock) by performing another simulation of e.g. 12 h;
- use *copy* again to take the end state of the (sub)network as initial state for the next simulation: as the other nodes are still disabled, their state will still be the one that was set previously;
- re-enable the rest of the network and generate another simulation of e.g. 5 days;
- the resulting graph will show the circadian clock resynchronizing to the changed alternance of the light/dark cycle in a few days’ time (see Fig. 4).

We are confident that further experiments similar to the ones described in [19] can be performed also in ANIMO, possibly adapting the proof of concept model to the more complex cases. Indeed, our work on a comprehensive ANIMO model encompassing both signal transduction and gene expression data in human chondrocytes [20–23] has shown that ANIMO can range from relatively simple models to realistic complex cases (whose presentation would fall outside of the scope of this paper).

A description of how the ANIMO model was built and how its data was compared to the data generated by the original ODE model is given in Additional file 1: Section B.1.

### Using ANIMO to generate hypotheses in human colon carcinoma cells

We now present a comparison with an existing fuzzy logic model, which we use also as an example of how ANIMO can be used to create reference models and help to obtain insight into complex biological events.

**Fig. 5:**
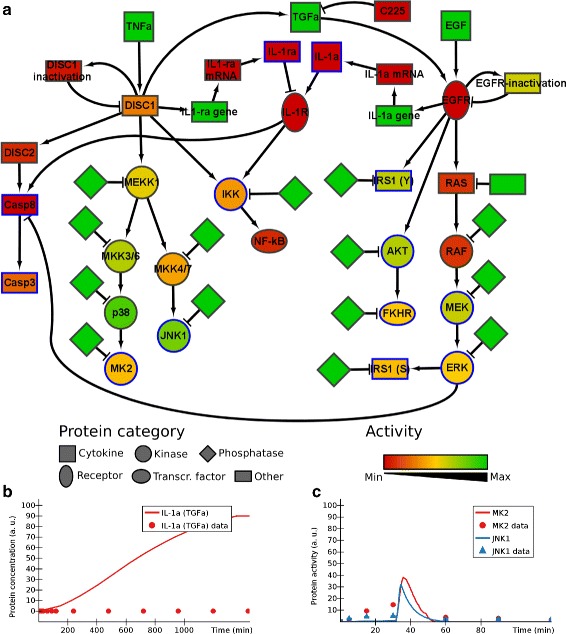
Signalling network downstream of TNF *α* and EGF in human colon carcinoma cells. **a** The model for the merged TNF *α* and EGF pathways. Node colours represent the activity level of the corresponding modelled reactants at time *t*=15 min after a stimulation of 100 ng/ml TNF *α* + 100 ng/ml EGF. **b** Modelled production of IL-1 *α* after stimulation with 100 ng/ml TGF *α* (24 h). **c** Modelled activation of JNK1 and MK2 after stimulation with 5 ng/ml TNF *α* + 10 *μ*g/ml C225 (2 hours). The _data suffix identifies experimental data; all other series are computed by ANIMO

We constructed a model of the signalling network downstream of TNF *α* and EGF in HT-29 human colon carcinoma cells, formalizing the crosstalk that takes place between the pathways at different levels of cellular regulation. We first modelled the two pathways in isolation (Additional file 1: Figures S7a, S8a, model available in Additional file 2), using information on protein interactions from the KEGG [24] and phosphosite [25] databases. These models were manually fitted to experimental data from previous studies [26, 27]. The models were mostly able to match the experimental data for the nodes included in either pathway (see Additional file 1: Figure S7b, c and S8b, c for some examples), but the crosstalk was not represented. For example, as MEK is only present downstream of EGF, the model contrasts with the experimental data by showing no activity of MEK following TNF *α* (called TNFa in the model) stimulation. To improve the model, we merged the two pathways into a single model and added the autocrine crosstalk between the pathways that has been described in [27]. Briefly, stimulation with TNF *α* leads to a rapid release of TGF *α* (TGFa in the model), which activates the EGF receptor (EGFR). This activation causes secretion of IL-1 *α* (IL-1a) at later time points. The effect of IL-1 *α* is down-regulated by the secretion of IL-1 receptor antagonist (IL-1ra) downstream of TNF *α*. The resulting model (Fig. 5a, model available in Additional file 2) was compared to the experimental data for treatments with 100 ng/ml TNF alone and 100 ng/ml EGF alone (see Additional file 1: Figures S9 and S10) [26].

At this point, the behaviour of the model deviated from the data for some of the nodes. Changing the parameters of the model, both manually and automatically (with the parameter sweep feature available in ANIMO [15], see Additional file 1: Section A.5 and Figure S4), was not enough to reproduce the behaviour shown by experimental data. This is an interesting situation, as it requires changes in the topology of the model [15], reflecting the formulation of a new hypothesis on the structure of the model. Below, we give two examples and show how adaptation of the model can be used to generate novel testable hypotheses.

**Fig. 6:**
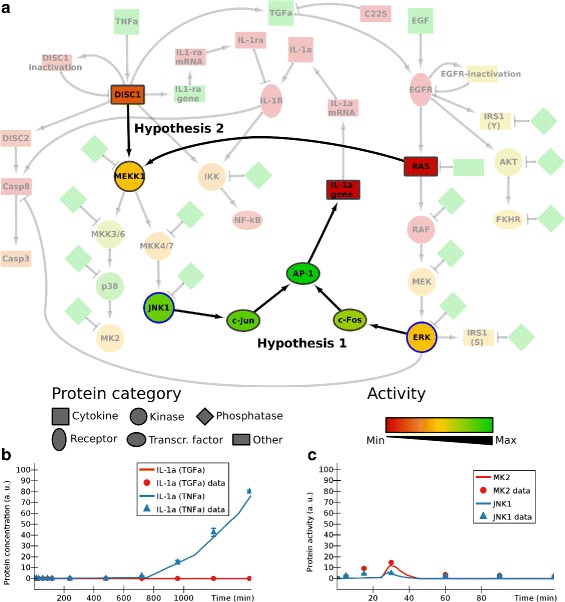
Signalling network downstream of TNF *α* and EGF in human colon carcinoma cells: improved model. **a** The model for the merged TNF *α* and EGF pathways after addition of the two hypotheses (*highlighted*). Hypothesis 1 assumes IL-1 *α* expression to depend on AP-1 activity, which in turn requires both c-Jun en c-Fos to be activated by JNK1 and ERK, respectively. Hypothesis 2 assumes RAS as an activator of MEKK1. Node colours represent the activity levels 15 minutes after stimulation of 100 ng/ml TNF *α* + 100 ng/ml EGF. **b** After the addition of the first hypothesis (activation of IL-1 *α* production depending both on JNK1 and ERK): production of IL-1 *α* after stimulation with 100 ng/ml TNF *α* (series IL-1a (TNFa)) compared with stimulation with 100 ng/ml TGF *α* (series IL-1a (TGFa)) (24 h). The IL-1a (TGFa) series is always 0. **c** After the addition of the second hypothesis (activation of MEKK1 downstream of EGFR): activation of JNK1 and MK2 after stimulation with 5 ng/ml TNF *α* + 10 *μ*g/ml C225 (2 hours). The JNK1 series is always 0. Additional file 1: Section B.3 explains how the dosage of 5 ng/ml TNF *α* was represented in the model. The _data suffix identifies experimental data; all other series are computed by ANIMO

Experimentally, treatment with TGF *α* alone does not lead to secretion of IL-1 *α*. Instead, a co-stimulation with TGF *α* and TNF *α* is required [27]. However, in the first version of the model, treatment with TGF *α* was sufficient for IL-1 *α* expression (Fig. 5b). Given the time delay until secretion of IL-1 *α*, it can be expected that *de novo* synthesis of IL-1 *α* is required and that both TNF *α* and TGF *α* are needed to activate transcription of the IL-1 *α* gene. JNK1 and ERK signal downstream of TNF *α* and TGF *α*, respectively, and are known to affect the activity of multiple transcription factors. We altered the model to make activation of IL-1 *α* expression dependent on both JNK1 activity and ERK activity (Additional file 1: Figure S11, edges linking JNK1 and ERK to IL-1a gene). After this modification to the model, IL-1 *α* was no longer secreted upon stimulation with TGF *α* alone, which greatly improved the fit between the measured IL-1 *α* levels and the model (Fig. 6b). This hypothesis could now be used to design a new experiment to validate IL-1 *α* as a target of combined JNK1 activity and ERK activity in HT-29 cells. For example, kinase inhibitors specific to JNK1 and ERK could be used to confirm that activity of both kinases is required for expression and secretion of IL-1 *α*. Performing the experiment is beyond the scope of this study, but this hypothesis finds support in literature: transcription factors c-Jun and c-Fos together form a heterodimer known as AP-1 and are activated by JNK1 and ERK, respectively [28, 29]. AP-1 has been reported to bind to the promoter of IL-1 *α*, providing evidence for a role in the regulation of IL-1 *α* expression [30]. Based on these findings in literature we included c-Jun and c-Fos in our model as transcriptional activators of IL-1 *α* (Fig. 6a, model available in Additional file 2).

As a second example, we considered the behaviour of JNK1 and MK2. In the model, both proteins were located downstream of TNF *α* but not TGF *α* or EGF. Hence, the model did not show an effect of C225, a pharmacological inhibitor of ligand-EGFR binding, on activation of JNK1 or MK2 after stimulation with TNF *α* (Fig. 5c). However, experimental data show that C225 strongly reduces activation of JNK1 and MK2 upon stimulation with TNF *α* [27]. This fact is indicative of a role for EGFR in activation of JNK1 and MK2. Since both JNK1 and MK2 are located downstream of MEKK1, we hypothesized that activation of MEKK1 is dependent on both TNF *α*-signalling and TGF *α*-signalling. In the model we added a new hypothetical node Hyp 2 (hypothesis 2) to link EGFR to MEKK1 (Additional file 1: Figure S11). This addition led to an improved fit of the model to the data upon treatment with TNF *α* + C225: activation of both MK2 and JNK1 was strongly suppressed by C225 (Fig. 6c). Stimulation with EGF alone did not lead to activation of JNK1 and MK2. These data support the validity of the modification to the model. Further support for a link between EGFR and MEKK1 was found in literature. Specifically, Ras has been reported as a direct activator of MEKK1 [31]. EGFR is a well-known and potent activator of Ras, which is why it was already in our network [24]. Other studies also report activation of JNK1 and phosphorylation of c-Jun downstream of Ras, which is consistent with an interaction between Ras and MEKK1 [29, 32]. Based on these findings, we adapted our model by removing the Hyp 2 node and creating a direct interaction between Ras and MEKK1 (Fig. 6a). Experimentally, the role of Ras could be confirmed by using a pharmacological inhibitor of Ras activity, and measuring the effect of this inhibitor on the activation of JNK1 and MK2. Together, our model suggests that EGFR activity is required but not sufficient for activation of JNK1 and MK2 in HT-29 cells.

There are other nodes for which the experimental data deviates from the model in one or more of the experimental conditions. A comparison between model and experimental data can be found in Additional file 1: Figures S12, S13 and S14. Comparing these results with the ones from [33] shows a better fit of the ANIMO model, which is also intrinsically more precise, being more mechanistic in nature (see Fig. 2). A complete deciphering of the signalling events in this biological system is outside the scope of this paper. Instead, we illustrated how interactive modelling of the dynamic behaviour of a signal transduction network can be used to extend previous pathway topologies and can lead to the generation of novel hypotheses.

## Discussion

### Final remarks on the models

We first described the construction of an ANIMO model of the circadian clock in *Drosophila Melanogaster*. This shows that the more abstract modeling paradigm of ANIMO is able to capture the dynamics of the regulatory network, leading to similar conclusions as an ODE model that had been published previously [19]. The biggest difference between the construction of these models is that the model in [19] is constructed on the basis of a detailed representation of the relevant biochemical reactions. ANIMO describes an abstract and aggregated view in terms of interactions, where the qualitative effect of each interaction is captured by a single parameter (see [11] for more details). In ANIMO a number of network nodes is drawn for the molecules involved. These nodes are then linked by directed interactions that represent cause-and-effect relationships. This abstract and graphical way makes it easier for biologists to create large networks in a compositional way: each node in the network can be disabled at any time by the user, or extra nodes can be added, without having to change any of the existing interactions. So ANIMO may yield models that are less complex than ODE models, possibly at the price of lower model precision: the curves representing oscillation of protein activities in the ANIMO model are not as precise as those obtained from the original ODE model (see Additional file 1: Figure S6).

We also showed the construction of an executable model of signalling events downstream of TNF *α* and EGF in human colon carcinoma cells. This data set has been used for previous modelling studies, based on partial least-squares regression and fuzzy logic [33, 34]. The partial least-squares regression model describes an abstract data-driven model that uses statistical correlations to relate signal transduction events to various cellular decisions. This type of modelling is very useful in uncovering new and unexpected relations. It is also successful in making predictions, but gives little direct insight in the dynamic behaviour of the network (see Fig. 2). Fuzzy logic analysis led to a model that gives a better fit to the dynamic network behaviour than discrete logic (Boolean) models. Inspection of the inputs to the logical gates that were used to model protein behaviour led to the prediction of novel interactions between proteins, showing the usefulness of this approach. For most of the proteins, such as JNK1, time was used as an input parameter. This means that the activity of some nodes at time point *t* was made dependent also on the value of *t* itself: thus, time becomes a variable in the model. For example, discretizing values in the two categories high and low, the logical gates “if TNF *α* is high*AND* time is low, then JNK1 is high” and “if TNF *α* is high*AND* time is high, then JNK1 is low” were used to describe the dynamic behaviour of JNK1. Although this leads to a representative description of the dynamic behaviour of JNK1, peaks in protein activity at early time points, as measured in wet-lab experiments, were not reproduced by the fuzzy logic model. Moreover, the fuzzy logic model gave no insight in the molecular interactions that are involved in activation or inhibition.

Here we used a data set based on the wet-lab experiments described in [26]. We used the resulting experimental data, together with knowledge from curated databases [24, 25] to construct an executable model of the biological system. In contrast to the two approaches described above (partial least-squares regression and fuzzy logic), ANIMO is aimed at the construction of more mechanistic models, mimicking biochemical interactions in silico. This way of modelling gives a different type of insight. In the process of model construction, we extended a prior-knowledge network with time-dependent extracellular crosstalk that has been reported previously [27]. To come up with possible explanations for a disagreement between the model and the experimental data, two additional layers of crosstalk were introduced, at the signal transduction and transcriptional level. These modifications improved the fit of the model to the data and can be interpreted as novel testable hypotheses. Finally, we proposed new experiments that could be carried out to test these hypotheses, closing the empirical cycle. Together, our model sheds more light on the intricate entanglement between the TNF *α* and EGF pathways at multiple cellular levels. But above all, the model provides an excellent starting point for further investigation.

### User experience: ANIMO and other modelling tools

Different formalisms are in use in the field of computational modelling of biological systems, each with their specific characteristics. Many of these formalisms have been implemented into software tools to support modelling efforts. To compare ANIMO with existing tools, we have selected a number of mathematical formalisms, each connected to a supporting tool. With an emphasis on the modelling process rather than the final model, and in an attempt to evaluate the degree of “interactivity” of these tools, we compared them on the basis of the following parameters:

1. **Domain-specific interface:** the underlying formalism is manipulated through an interface targeted towards the biological domain
2. **Visual modelling:** the tool allows the user to model using a visual interface, and is not exclusively founded on formula-, text- or table-based input forms
3. **Qualitative parameters:** parameters for reactions can be input as approximated estimations, and not exclusively as numbers
4. **Tight coupling with topology:** models are tightly and clearly coupled to the networks they represent, showing the visual representation of the model in a shape similar or comparable to the representation currently used by biologists for signalling pathways
5. **User-chosen granularity:** if discretization is applied during the modelling process, the user can change the granularity with which such discretization is made, possibly for each component of the model separately

Table 1 shows the comparison between ANIMO and the selected tools. The tools are grouped by underlying formalism, following the ordering of Fig. 2. The comparison encompasses no tools using statistical methods, as we concentrate on tools that allow to define the dynamics of biological networks from a more mechanistic point of view.

**Table 1:** Comparison between ANIMO and some existing approaches to modelling biological systems

Tool	Formalism	Domain-specific interface	Visual modeling	Qualitative parameters	Tight coupling with topology	User-chosen granularity
GINsim [40]	Boolean Networks	Yes	Yes	Yes	Yes	Yes ^a^
BooleSim [41]	Boolean Networks	Yes	Yes	No	No	No
CytoCopteR [35]	Fuzzy logic ^b^	Yes	Yes	Yes	Yes	Yes ^c^
ANIMO [10]	Timed Automata	Yes	Yes	Yes	Yes	Yes
Odefy [42]	Logic-based ODE	No	Yes ^d^	Yes	No	No
COPASI [43]	ODE, stochastic models	No ^e^	No	No	No	No
CellDesigner [44]	ODE	Yes	Yes	No	Yes	No
GNA [45]	PLDE	Yes	Yes	Yes	Yes	Yes ^a^
Virtual Cell [46]	ODE, PDE, stochastic models	Yes	Yes	No	Yes	No
Bio-PEPA Workbench [47]	Bio-PEPA	No	No	No	No	Yes
COSBI LAB [48]	BlenX	Yes	Yes	No	Yes	No
Cell Illustrator [49]	Petri Nets	Yes	Yes	No	Yes	No

A “Yes” under a column indicates that the modelling tool (mostly) fulfils the parameter, “No” indicates very limited or no fulfilment

^a^The user can choose the number of levels for each reactant, allowing to define multi-level models based on Boolean reaction dynamics

^b^Boolean logic and logic-based ODE models are also available

^c^The choice for the type of logic to be used determines also the granularity of the model

^d^Only if coupled with the yEd [50] graph editor

^e^While visual network modeling is absent, the MultiState Model Builder (MSMB [51]) editor provides an interactive support during the phase of model definition

Among related work, we would like to highlight the powerful tool CellNOpt [35]. CellNOpt is a software which can work with logic descriptions (Boolean, fuzzy) and differential equations, and automatically suggests the best network topologies to match a given data set. Thanks to the CytoCopteR plug-in for Cytoscape [36], which provides an accessible user interface, CellNOpt can be used in tandem with ANIMO: after computing the most likely network topologies with CytoCopteR, the biologist can carry on the analysis process with ANIMO, working on new hypotheses to explain the experimental data. Please note that this workflow is currently not implemented in a user-friendly way, and in order to perform it both CytoCopteR and ANIMO need to be installed. It is also possible to import SBML qual [37] models thanks to CytoCopteR’s import function and use them in ANIMO, as the basic properties of nodes and edges are automatically inferred. However, the *k* parameters of interactions as well as initial activities of nodes are set to default levels, and the user may need to change some of them in order to obtain a working network. We plan to extend ANIMO in such a way that the integration with CytoCopteR and other tools is made as smooth as possible. Full support of widely used model formats such as SBML qual will improve ANIMO’s interoperability, and this will positively reflect on the user experience.

Going beyond the user interface, there are a number of “pros and cons” for using ANIMO and Timed Automata in the biological context. First and foremost, as Timed Automata is an executable formal language, a state space can be derived from a Timed Automata model. This means that state space-related analyses such as model checking can be performed on Timed Automata: this can be done directly in ANIMO, as ANIMO acts as an intermediary towards the powerful model checking tool UPPAAL.

While ANIMO does not require the user to know Timed Automata or UPPAAL, it is necessary to possess some biological knowledge in order to build useful models. In particular, estimating activity rates may present difficulties that can be reduced with the help of biological intuition. As an example, consider the difference in rate between the production of a protein and a post-translational modification such as phosphorylation: biological knowledge leads to choose a (much) lower rate for the former than for the latter. A second example, and an additional rationale for the development of tools like ANIMO that put the biologist in charge, is the translation of experimental numerical data into activity levels. It is necessary to have semi-quantitative data as reference [11] (the data used as reference in the TNF *α*-EGF model is mostly based on semi-quantitative western blot experiments [26]), together with biological knowledge to define a sensible correspondence between experimental values and activity levels.

For each reactant modelled in ANIMO we assume that the total amount of active and inactive molecules remains constant. While this assumption is not always applicable, it encourages abstract thought: many biological processes can be represented as networks driven by *activity-based* interactions (see Methods section). Even if with a limited scope, ANIMO can be applied also in the analysis of metabolic processes, using activity to represent concentration as proposed in the Background section. While it cannot be expected from such models to be a completely realistic representation of their target biological processes, they can still be a useful tool. This can be seen for example in the circadian clock model in the Results section, where mRNA and protein concentrations were abstracted to activity-driven processes in the ANIMO model.

Finally, a note on the performance: the interactive use for which ANIMO is conceived implies that model analysis should require an amount of time small enough to encourage the user to experiment with different model configurations. The simulation of an ANIMO model is not computationally expensive, requiring minimal amounts of memory and CPU time. For example, on an Intel®; Core™ i7 CPU working at 2.80 GHz, computing a 24-h simulation run of the model in Fig. 3a takes about 0.69 sec while the model in Fig. 6a takes about 1.14 sec. The larger model on which we are working [22, 23] is an order of magnitude larger than the ones presented in this paper (92 nodes and 123 interactions), and in that case computing a simulation where the state of the network dramatically changes (nearly all nodes undergo significant activity variations) takes about 16 sec. All the averages were computed based on 100 simulation runs.

## Conclusions

In this paper we discussed the placement of ANIMO among other modelling paradigms and tools, highlighting ANIMO’s strong points.

From the point of view of model precision, we position ANIMO between fuzzy logic and ODEs. Being less parameter-intensive than ODEs and more precise than logic-based models, ANIMO models are useful for a wide range of applications.

ANIMO adds a dynamic component to the static networks already familiar to biologists, allowing the domain experts to build formal executable models of complex biological networks. ANIMO is not the first tool to provide an interface to a modelling formalism: as shown in Table 1, such interfaces exist in many other tools. Focusing on user-friendliness and interactive modelling, ANIMO makes computational modelling more accessible to experts in biology. Thanks to the visual interface provided by Cytoscape, networks are represented according to biological conventions. Model parameters are kept to a minimum and can be directly accessed by mouse-clicking on nodes and edges. Because of the automatic translation of the network topology and user-defined parameters into an underlying formal model, training in the use of formal methods is not needed.

## Methods

### Modelling biological interactions with timed automata

**Fig. 7:**
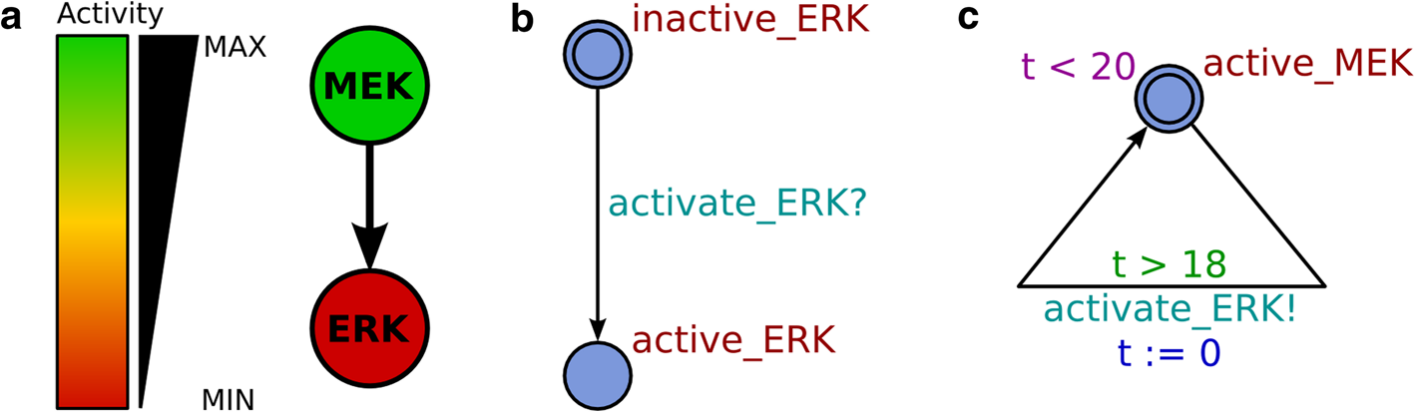
Abstraction of a biochemical reaction to a Timed Automata model. **a** Classical depiction of a well-studied intracellular signal transduction reaction: protein MAPK-ERK kinase (MEK) activates downstream protein extracellular-regulated kinase (ERK). **b** A Timed Automaton model of ERK, consisting of two locations (*circles*), inactive_ERK and active_ERK, and one transition (*edge*) between the locations. This transition will take place when it is possible to synchronize with the corresponding action activate_ERK! in the MEK automaton. **c** A Timed Automaton model of active MEK, consisting of one location and one transition. t<20 is called an invariant on the location, allowing residence in this location as long as clock time t is smaller than 20 units. t>18 is called a guard on the transition, allowing the transition to take place when clock t is greater than 18 units. Together, the invariant and guard in this example ensure that the transition must take place in the (continuous) time interval 18<t<20. When the transition takes place, the action activate_Erk! is performed (thus allowing the ERK automaton to reach the active_ERK location) and the local clock coupled to this automaton is reset, t:=0

Timed Automata have been shown to be a powerful formalism to model biological processes [17, 38, 39]. A timed automaton consists of locations and transitions between these locations (see Fig. 7), and a system of timed automata can be used to model a system of interacting molecules. At any time, each automaton is in a specific location, and together these locations represent the current state of the biological system. Each timed automaton can have one or more clocks associated to it, allowing temporal control of transitions between locations. The transitions are used to represent interactions between molecules. Fast interactions take less time than slow interactions to perform an activation or inhibition step. We have previously described in detail how approximated reaction kinetics [10] can be used to calculate the timing of molecular interactions (see also Additional file 1: Section A.1). Figure 7 presents a small example that illustrates the basic properties of Timed Automata. This model describes the activation of ERK by MEK.

### Example: building a model based on data

To illustrate the use of ANIMO in a practical environment, we will demonstrate the generation of a basic version of the model described in the Results section. The model is based on a literature compendium of signal transduction events in HT-29 human colon carcinoma cells [26]. This data set comprises triplicate measurements of 11 different protein activities or post-translational modification states at 13 time points after treatment with different combinations of tumour necrosis factor- *α* (TNF *α*), epidermal growth factor (EGF) and insulin. The data set contains relative protein levels and activities, and no absolute quantities, which is the typical situation in biochemistry. To start, we normalized measurements for each protein to the maximum value in the complete experiment, resulting in a nondimensionalized data set that is suitable for use with ANIMO (see Additional file 1: Section B.2).

**Fig. 8:**
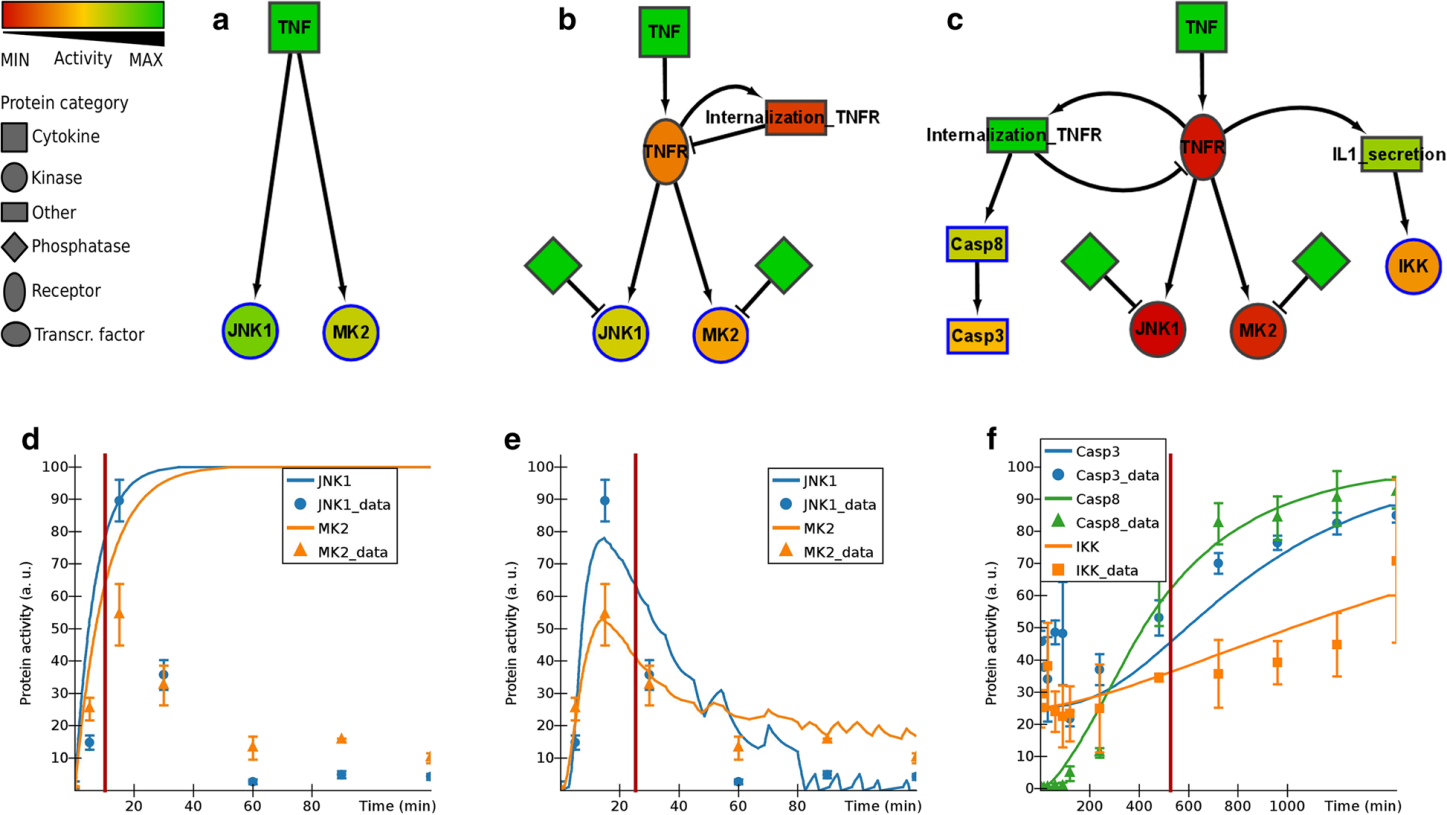
Construction of an ANIMO model of signal transduction events in human colon carcinoma cells upon stimulation with 100 ng/ml TNF *α*. Graphs below show the dynamic behaviour of the corresponding models above, comparing it to the measured activity values from [26] (error bars represent the standard deviation). On the vertical axis, “100” represents the maximum protein activity in the complete experiment. A red vertical line in each graph highlights an arbitrary time point in the time course: nodes in the corresponding model are coloured according to their activity at that time point. **a**, **d** Basic model showing direct activation of JNK1 and MK2 by TNF *α*. No peak dynamics are observed because no inactivating processes are present. **b**, **e** The model after addition of inactivating phosphatases and a negative feedback loop that down-regulates TNFR. Note that adding TNFR internalization or phosphatases alone would not be enough to reproduce activity peaks. **c**, **f** The model after addition of IKK, IL1-secretion (abstracting the autocrine IL-1 signalling described in [27]), Casp8 and Casp3, showing the late response to TNF *α* signalling. As the data set did not contain values for cleaved caspase-3, but only for its non-cleaved precursor pro-caspase-3, we computed the Casp3_data series as 100 *%*−[pro-Casp3]

In Fig. 8 we show the stepwise construction of a model of a small part of the network that is able to account for measured variations in activity of inhibitor of nuclear factor kappa-B kinase (IKK), c-Jun N-terminal kinase-1 (JNK1), mitogen-activated protein kinase-activated protein kinase 2 (MK2), Caspase 8 (casp-8) and Caspase 3 (casp-3) upon stimulation with 100 ng/ml TNF *α*. In this example we aimed for inclusion of a minimum number of nodes in the network, while preserving biological relationships. Multi-step cascades were aggregated into a single step when possible. Parameters for all reactions were set manually, resulting in a close match between the model and the patterns observed in the dataset.

## Abbreviations

Table S1 in the Additional file 1 contains explanations for the abbreviations used in the paper, including UniProt IDs (when applicable)

## Additional files

Additional file 1 Additional material on ANIMO and Timed Automata, Notes on the models presented in the Results section, Naming conventions used in the paper, Supplementary figures. (PDF 7270 kb)

Additional file 2 ANIMOmodels and data for the case studies. (ZIP 152 kb)
