# Supplementary material for: Modelling with ANIMO: between fuzzy logic and differential equations
Source: BMC Syst Biol. 2016 Jul 27;10:56. doi: 10.1186/s12918-016-0286-z (PMC4962523; doi:10.1186/s12918-016-0286-z)
Supplement: Additional file 1 — Additional material on ANIMO and Timed Automata, Notes on the models presented in the Results section, Naming conventions used in the paper, Supplementary figures. (PDF 7270 kb) [file 12918_2016_286_MOESM1_ESM.pdf]

Additional Materials

Table of Contents

A ANIMO and Timed Automata **1**

A.1 Modelling abstractions 1

A.2 ANIMO 2

A.3 Timed Automata model 2

A.4 Granularity of an ANIMO network node 3

A.5 Choosing the parameters for an ANIMO model 3

B Additional notes **7**

B.1 ANIMO model of the Drosophila circadian clock 7

B.2 Normalizing experimental data for use with ANIMO 10

B.3 Note on the parameters in the TNFα-EGF model 10

C Naming conventions **11**

D Supplementary figures **13**

Appendix A: ANIMO and Timed Automata

A.1 Modelling abstractions

In living cells, cascades of chemical and physical interactions enable propagation of signals through molecular networks. In this process, the activity of upstream molecules induces a change in the concentration or activity of downstream molecules. For many reactions, the values of the kinetic parameters are unknown or difficult to collect. This lack of knowledge hampers the feasibility of computational models that describe molecular networks in fine mechanistic detail, especially for larger networks. As a solution to this problem, we propose the construction of models at a higher level of abstraction, thereby reducing the number of parameters involved. In choosing a suitable abstraction level, it is important to retain enough descriptive power to give a meaningful formal description of the topology and the associated dynamic behaviour of biological networks.

As a first abstraction in ANIMO models, the active and inactive forms of each network component are represented together by a single node in the network. Each of these nodes is characterized by its *activity level*, which represents the fraction of active molecules of that molecular species. When a molecule is known to be constitutively active, changes in concentrations of that molecule are treated as changes in its activity level. Activity levels are discretized into integer variables with a user-defined granularity, ranging from Boolean (2 levels) to near-continuous (100 levels).

Detailed biochemical reaction mechanisms are abstracted to *interactions*, which can represent either activations ($\rightarrow$) or inhibitions ($\dashv$). This aggregation of elementary reactions into single interaction steps reduces the number of kinetic parameters involved, while preserving cause-and-effect relationships. For example, consider a reaction in which enzyme E phosphorylates and activates substrate S, transferring a phosphate group from a molecule of ATP to a molecule of S. Biochemically, this reaction can be represented as

$$E + S + ATP \rightleftarrows ES + ATP \rightarrow ES^P + ADP \rightleftarrows E + S^P + ADP,$$

with conservation condition $S + S^P =$ constant and $ATP + ADP =$ constant. Under the assumption of ATP constantly being replenished by the cell, this reaction is abstracted in ANIMO to the corresponding interaction

$$E \rightarrow S.$$

Each occurrence of the interaction $E \rightarrow S$ will increase the activity level of S by one discrete step. Since the activity level is defined as the active fraction of a molecular species, an increase in the active fraction implies a decrease in the inactive fraction. Hence, the original conservation condition is automatically satisfied. The interaction rate, R, depends on the activity levels of the reactants involved and on a single kinetic parameter k that is set by the user. The three available interaction scenarios can be interpreted as abstracted kinetic rate laws:

1 $R = k \times [E]$: the interaction rate depends only on the activity level of the upstream node.

2 $R = k \times [E] \times [1 - S]$ (activations) or $R = k \times [E] \times [S]$ (inhibitions): the rate depends on the activity levels of both the upstream and downstream participants. Activations depend on the presence of inactive substrate, *[1 - S]*, whereas inhibitions depend on the level of active substrate, *[S]*.

3 $R = k \times [E_1] \times [E_2]$: this scenario can be used when the activation or inhibition of a downstream node depends on the simultaneous activity of two upstream nodes. This scenario is comparable to an *AND-gate* in Boolean logic.

As we have shown in the Results Section and in [10, 11, 15], the abstraction described here preserves ample expressivity to capture the dynamic behaviour of a biological network.

A.2 ANIMO

The modelling approach described in Section A.1 and in the Methods Section is implemented in the software tool ANIMO (Analysis of Networks with Interactive MOdelling, [10]) as a plug-in to the network visualization tool Cytoscape [12]. The visual interface of Cytoscape makes the construction, expansion and rewiring of a network topology a fast and user-friendly process.

When a new node is added to the network, it has to be initialized with the number of activity levels and its initial activity. For each interaction, a scenario needs to be selected, together with the corresponding kinetic parameter and the interaction type: activation or inhibition. All settings can be readily adapted by double clicking, or via a table of nodes or interactions.

ANIMO automatically translates the user input to a TA model, which is then simulated with the model checking tool UPPAAL [14]. The results are subsequently parsed and translated to a graph that shows the dynamic behaviour of nodes in the network. A schematic overview of this process is given in Figure S1. No training or prior knowledge on the use of TA or UPPAAL is needed in order to benefit from ANIMO. Nevertheless, the TA model and the model checking process in UPPAAL can be accessed when desired by the user.

The dynamic behaviour of a model can be interactively explored by moving a time slider underneath the graph to highlight time points in a simulation. In the network view, each node will be coloured according to its activity level at the selected time point. Experimental data can be compared to the model by importing and superposing these data upon an output graph from the model (all the graphs in this paper were made directly in ANIMO). The ANIMO user workflow and the features described above are illustrated in a video on ANIMO's web page `http://fmt.cs.utwente.nl/tools/animo`.

A.3 Timed Automata model

The Timed Automata (TA) model underlying an ANIMO network is generated whenever an analysis is requested by the user. Starting from the network represented in the Cytoscape-based user interface, ANIMO automatically generates a TA model to be used with UPPAAL. The analysis result is then parsed and properly presented to the user, for example as a graph of reactant activity levels. This workflow is described in Figure S1.

Each TA model generated by ANIMO contains one automaton for each interaction (activation or inhibition) in the network. A TA representing an interaction

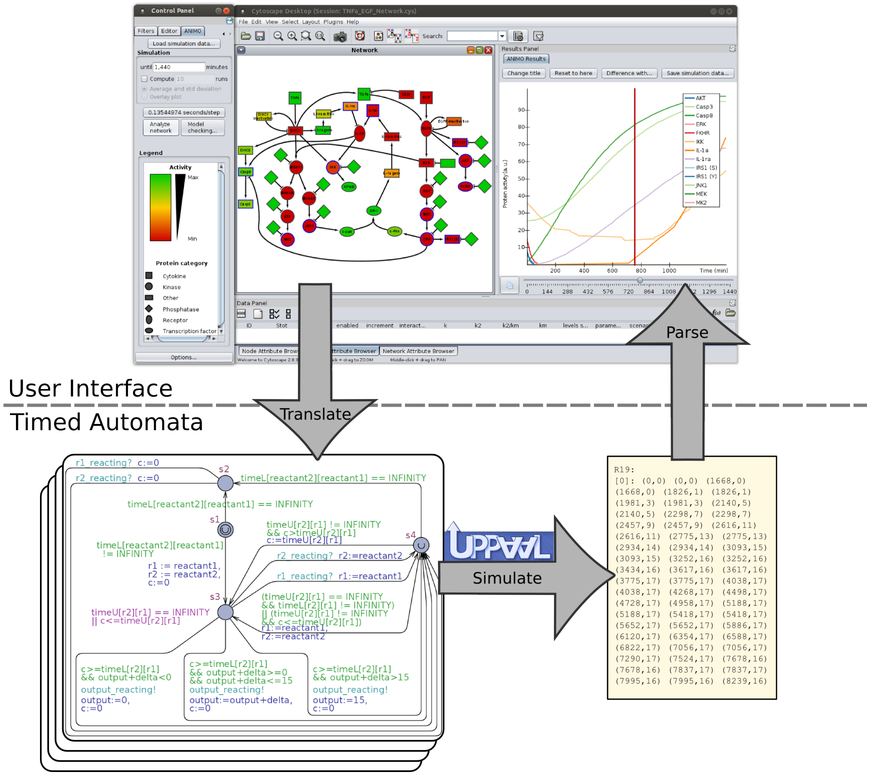

Figure S1: The passages intercurring between the press of the *Analyse network* button and the generation of an activity level graph. The model represented in the Cytoscape visual interface is automatically translated by ANIMO into a network of Timed Automata, which is then simulated using UPPAAL. A simulation produces, for each selected node, a series of pairs (t, a), with t a time instant along the simulation and a an activity level. These data are then parsed and represented as a graph, which is shown in ANIMO's user interface.

performs a cyclic series of steps, continuously updating the target of the interaction it represents, and adapting the timing of the next update according to the user-defined dynamics. Synchronizations between different automata occur when the activity level of a network component (e.g. ERK) changes: this allows the automata depending on that component to update their time settings.

The abstract behaviour of the interaction MEK → ERK in the TA model used in ANIMO is described in Figure S2. There, the activity levels of MEK and ERK are represented by variables called, respectively, $\text{MEK}_{\text{activity}}$ and $\text{ERK}_{\text{activity}}$. A more detailed description of the TA model underlying ANIMO was presented in the IEEE Journal of Biomedical and Health Informatics [10].

A.4 Granularity of an ANIMO network node

Figure S3 shows the differences between different choices for the number of levels of a node. This allows to adapt a model to the quality of experimental data. Also, lowering granularity will increase the computational performances: this will be particularly noticeable in very large models (hundreds of nodes).

A.5 Choosing the parameters for an ANIMO model

Figure S4, taken from [15], represents a workflow we suggest to follow when choosing the parameters for an ANIMO model. We briefly describe the steps here:

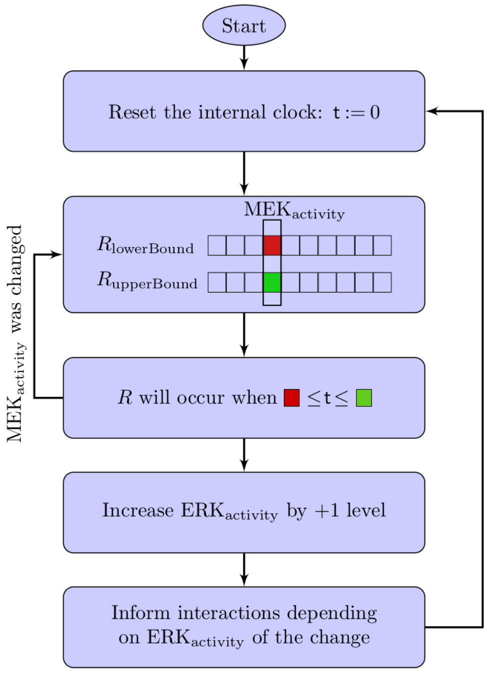

Figure S2: Schematic overview of the steps taken during a simulation run by a Timed Automaton modelling an interaction R that increases ERK$_{\text{activity}}$ and depends only on MEK$_{\text{activity}}$. In this example, MEK is represented with a granularity of 10 activity levels. After resetting the internal clock t, the automaton sets the time constraints for the interaction. MEK$_{\text{activity}}$ is used as the index inside the time tables $R_{\text{lowerBound}}$ and $R_{\text{upperBound}}$, which contain pre-computed lower- and upper-bounds for the interaction timing. Once the bounds have been identified, R can occur when t reaches a value inside the continuous time interval [■,■]. When it occurs, R increases the value of ERK$_{\text{activity}}$ by 1. All interactions that depend on ERK$_{\text{activity}}$ are notified of the change (via a synchronization on a specific channel), so that the associated time bounds are updated accordingly. After resetting the clock t, the process can restart. If MEK$_{\text{activity}}$ was changed by another automaton before the occurrence of R, the time bounds are updated according to the new activity level of MEK.

1 Define a starting topology, based on literature data and current knowledge on the network.

2 Choose the interaction parameters from the pre-set qualitative values, based on experience (e.g., protein expression is much slower than phosphorylation). The choice on the approximated kinetics can be for the most part based on scenario 1, referring to Section A.1.

3 Check that the network behaves as expected (e.g. addition of EGF makes ERK activity rapidly increase and successively decrease).

4 Possibly iterate the steps 1 to 3 until the network behaves as expected.

5 Compare the model with experimental data.

6 If the model does not fit the data, change the parameters using more precise numerical values and/or scenario settings.

7 If the data fitting is still not satisfactory, apply parameter sweep to (subsets of) the model as described in [15]. Briefly, multiple simulations will be automatically computed, choosing for each parameter k a different value in a user-defined (linear- or logarithmic-scale) interval. The parameter choices generating the results that best match the reference experimental data will be displayed to the user.

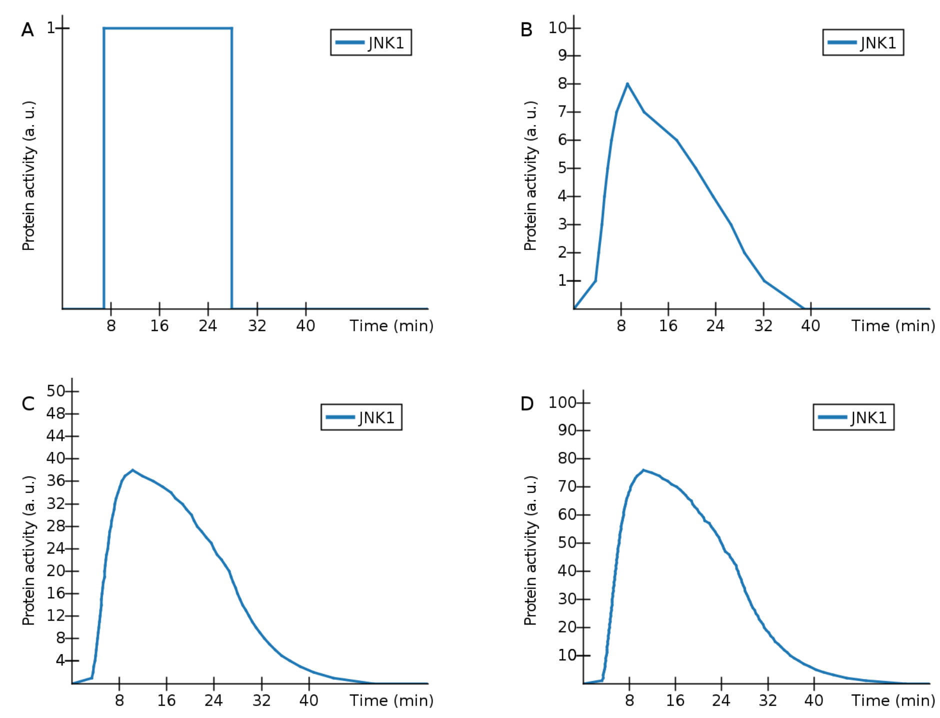

Figure S3: Comparing different reactant granularity settings. (A) 2 levels, **(B)** 10 levels, **(C)** 50 levels, **(D)** 100 levels. The JNK1 series is computed from the model presented in Figure 6, considering 100 ng/ml TNFα as treatment condition over a period of 60 minutes.

8 When a good fit is found, add non-determinism to the model and ensure that the behavior does not change significantly under reasonably high uncertainty settings (i.e. perform a sort of sensitivity analysis).

9 If the model can fit the data only with very precise parameter settings, or a parameter configuration that fits the data cannot be found after a reasonably extensive search on the parameter space, change the network topology possibly adding more nodes from literature. If no candidates can be found in literature, speculative candidates can be found by "playing" with the current configuration in ANIMO's user interface. We advise to let expert biologists perform this step on their own, or to perform it under their supervision, in order to keep the network both simple and realistic. For the same reason, we also advise not to introduce more than a couple of nodes at a time.

10 Parameters for newly introduced interactions can be chosen as in step 2, possibly iterating steps 2 to 7 to refine the parameters.

11 To check how changes in the network are reflected in the behavior of the model, we advise to use the comparison tools described in ANIMO's user manual [16] (see in particular the use of "Reset to here" and "Difference with...") and in [15].

If the model fits the data reasonably well only after modifications to the initial topology, it may contain some hypothetical or unexpected nodes (see the Results section in the main text). In that case, additional laboratory experiments can be designed starting from the hypotheses introduced in the model, and help the biologists decide which are the most precise explanations for the experimental observations.

A simplified graphical representation of the workflow can be found in Figure S4: the three degrees of precision with the parameter settings are highlighted with progressively darker shades of blue. Moreover, the figure helps to stress the fact that in our method changes in topology (green boxes) should be made as a last step, after a reasonable attempt at making the model fit via parameter adjustments. Again, it is important to remember that if a model behaves correctly only for very narrow parameter settings then it may not be as robust as expected, so a review of its topology may be needed.

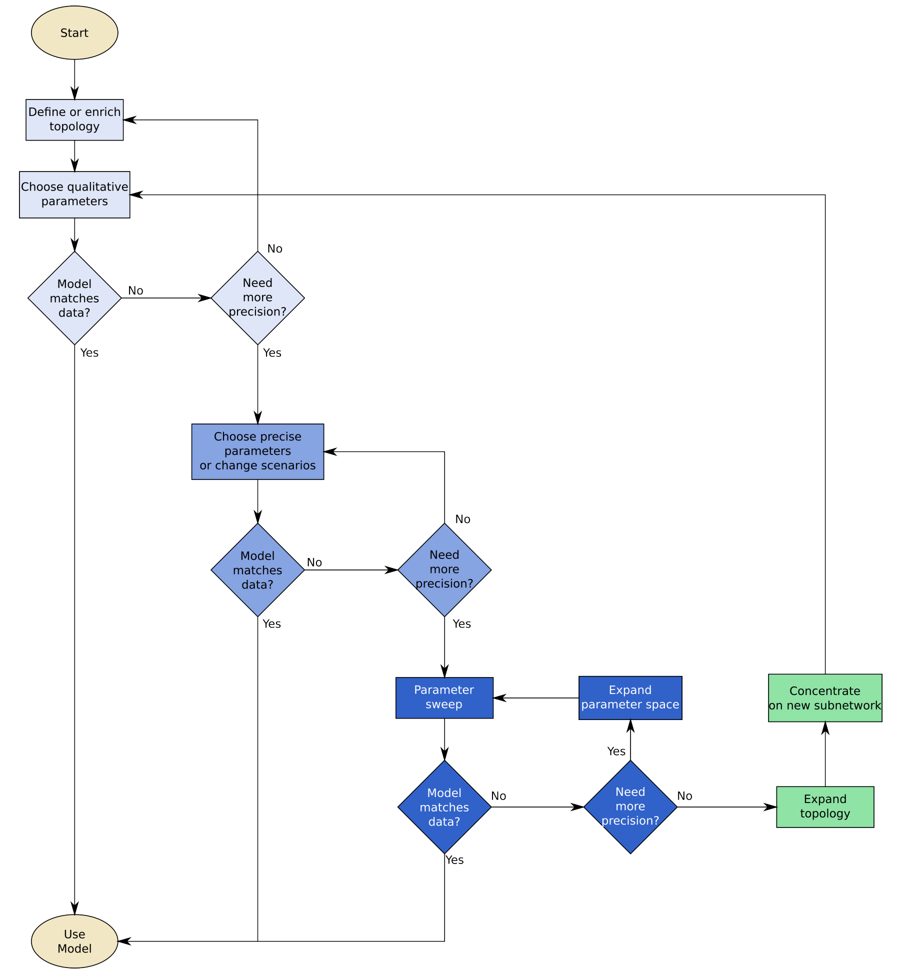

Figure S4: ANIMO modeling workflow.

Appendix B: Additional notes

B.1 ANIMO model of the Drosophila circadian clock

The topology of the model we built in ANIMO to represent the circadian clock of *Drosophila Melanogaster* was presented in Figure 3A and is the same as the one presented in [19]. The parameters were also initially taken from the original model, mapping them in the following way:

- $k(i \dashv i) = r_i$
- $k(i \rightarrow j) = T_{i,j}$

where $k(i \dashv i)$ is the k value for the self-inhibition of reactant i in the ANIMO model, r_i is the *maximal rate of formation* of reactant i in the ODE model, $k(i \rightarrow j)$ is the k value for the interaction between reactants i and j in the ANIMO model, and $T_{i,j}$ is the *regulatory weight* of the same interaction in the ODE model. All approximation scenarios were set to Scenario 1. We note that the mapping described here is relatively straightforward mainly because the original ODE model was already founded on a higher abstraction level, and thus used less parameters, than most ODE models. It is more common for ODE models to be closer to the actual biochemical description of reactions: translating such models into ANIMO would require the user to abstract away some details. However, ANIMO was specifically built to be used by expert biologists, and biology researchers possess a high level of understanding of biochemical reactions: this makes the aforementioned abstraction a less time-intensive task.

In order to model the alternance of day and night that makes the concentration of CRY oscillate, we used a repressilator-like subnetwork [13] (Fig. S5), which was abstractly represented in Figure 3A by the node labelled Day/Night. The alternance of day and night was represented in the original ODE model as a piecewise linear approximation of experimental data.

The described setting for the network topology and parameters led to 24-hour oscillations that were consistent with the ODE model, but the amplitude of oscillation for some nodes was not easily noticeable: they were oscillating by less than 5 activity levels. This ANIMO model we initially obtained was also translated to an ODE model in UPPAAL [51][1] and simulated: it showed the same oscillations as the ANIMO model. This let us exclude that the oscillations initially observed were due to a modelling artefact. At this point, the interaction parameters in the ANIMO model were modified by hand in order to increase the amplitude of the less noticeable oscillations. While applying these modifications, the behaviour of the ANIMO model was kept as close as possible to the original ODE model.

The ANIMO model we finally obtained was then simulated for a 24-hour period, and its results compared to the original ODE model (see Figures 3B-E and S6). In this comparison, both models were taken "as is", without altering their initial conditions nor adding/removing components. Such alterations were done when performing specific experiments as dicussed in the Results section.

[1] In particular, our model was built similarly to the model described in Section 6 of [51] (Biological Oscillator). The support for hybrid systems in UPPAAL was used in order to simulate a system of ordinary differential equations (thus a hybrid system where no discrete events occur).

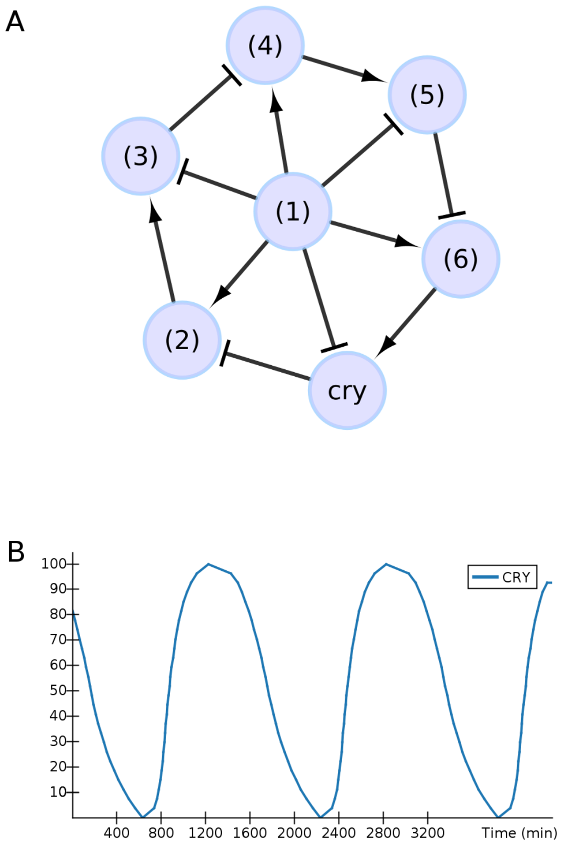

Figure S5: Modelling the day/night cycle in ANIMO.(A) The repressilator-like subnetwork used to represent the alternation between day and night that cause the oscillations in CRY concentrations in the network modelled in the Results Section. The repressilator-like subnetwork affects the CRY node in the ANIMO model through the interaction cry → CRY. **(B)** A graph plotting the oscillations in CRY along a period of three days.

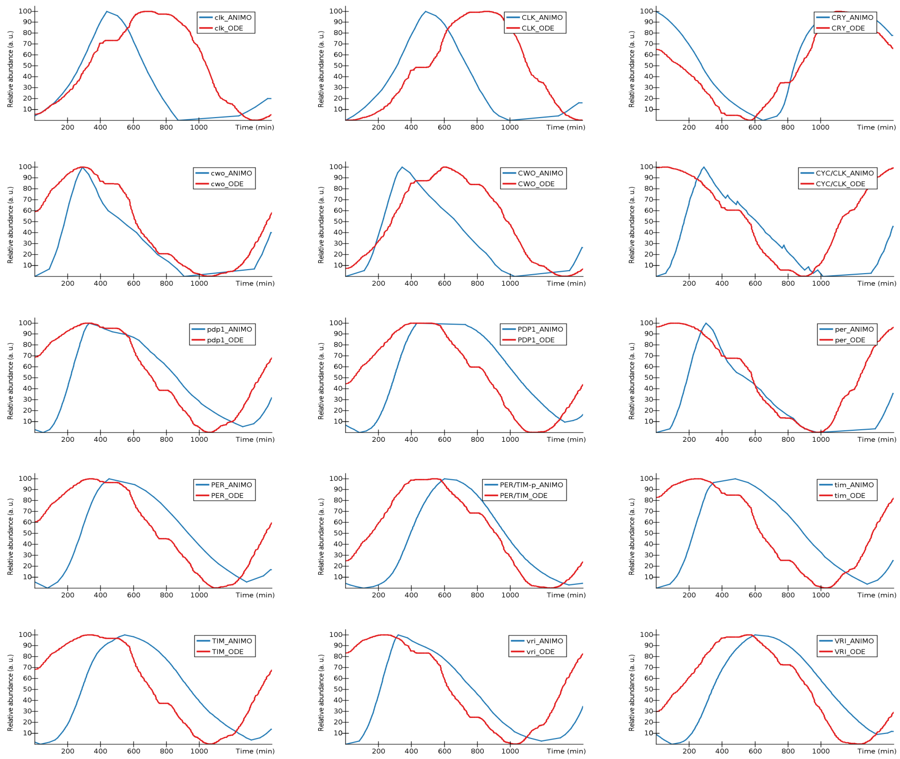

Figure S6: Comparing the simulation results from the ANIMO and ODE models of *Drosophila Melanogaster* circadian clock. Blue lines (_ANIMO series) represent data from the ANIMO model in Figure 3, while red lines (_ODE series) represent data from the ODE model in [19].
The raw data coming from the two models were aligned to have a roughly close initial point, and all amplitudes were normalized following the procedure described in Suppl. Sect. B.2.

B.2 Normalizing experimental data for use with ANIMO

Document S1 in the Supplemental Data of [27] contains three tables, named Replicates, Averages and DPLSR dataset. The data we use to compare the results computed with ANIMO are based on the values from the Averages table. In particular, we compute activity data by performing a normalization on a $0 \ldots 100$ scale using this formula

$$v_{\text{norm}} = \frac{v}{v_{\max}} \times 100$$

where v is the datum to be normalized taken from the column v in the Averages table, v_{norm} is the normalized value and $v_{\max}$ is the maximum value over the whole column v in the Replicates table. For each series, we compute also the standard deviation using the triplicate measurements present in table Replicates. The standard deviation is also normalized using the formula presented for the average. Each table produced with this process contains a subset of the columns from the Averages table, and refers to one treatment condition only. A column with time references is added to the table in first position. Finally, a column named *Number_of_levels* containing only the value 100 (see the instructions in the ANIMO manual available online at `http://fmt.cs.utwente.nl/tools/animo/content/Manual.pdf`) is added at the rightmost position. The tables are all exported in .csv format to be used with ANIMO, and are included in the *Model_and_data.zip* file in the additional materials of the present work.

B.3 Note on the parameters in the TNFα-EGF model

The parameters in the models in Figures 5 and 6 have been set by fitting the model to the experimental data for the treatment conditions with 100 ng/ml TNFα, 100 ng/ml EGF and 100 ng/ml TNFα + 100 ng/ml EGF. In the model we have set the activity of the corresponding TNFa and EGF nodes either at their maximum value or at 0, depending on the modelled treatment. This level is a dimensionless quantity that indicates either the maximum or minimum activity level in the data set. However, we found that in order to model the condition with 5 ng/ml TNFα we needed to set the activity of the TNFa node to a value higher than 5 out of 100. We believe that this has to do with the fact that 100 ng/ml is a highly supra-physiological concentration of TNFα, that will rapidly cause activation of all receptors present. Fitting the model to this experimental condition may have resulted in slight deviations in the parameter values. Nevertheless, the modelling results illustrate that building a model with basic kinetic rate laws can give useful predictions over a range of concentrations. Figure 5C was obtained using the model in Figure 5A and setting the activity of TNFa to 10/100 (lower values gave 0 activity for both JNK1 and MK2), while Figure 6C was obtained from the model in Figure 6A with TNFa set at 6/100. In both cases, C225 was set at 100/100.

Appendix C: Naming conventions

Table S1 explains the abbreviations used in the paper.

Table S1: Explanation of the abbreviated names referring to molecular species in the main text.

Abbreviation	Full name	UniProt ID
Akt	protein kinase B	P31749
AP-1	activator protein 1	heterodimer of c-Jun and c-Fos
Casp3	caspase 3	P42574
Casp8	caspase 8	Q14790
c-Fos	proto-oncogene-protein c-fos	P01100
c-Jun	Jun activation domain binding protein	P05412
CLK	clock	O61735
CRY	cryptochrome	O77059
CWO	clockwork orange	Q9VGZ5
CYC	cycle	O61734
CYC/CLK	cycle-clock complex	
DBT	double-time kinase	O76324
DISC1	death-inducing signaling complex 1	
DISC2	death-inducing signaling complex 2	
EGF	epidermal growth factor	P01133
EGFR	EGF receptor	P00533
ERK	extracellular regulated kinase	P27361
FKHR	forkhead box protein O1	Q12778
IKK	inhibitor of nuclear factor kappa-B kinase	O14920
IL-1a	interleukin 1 α	P01583
IL-1R	interleukin 1 receptor	P14778
IL-1ra	interleukin 1 receptor antagonist	P18510
IRS1 (S)	insulin receptor substrate 1 (Serine 636)	P35568
IRS1 (Y)	insulin receptor substrate 1 (Tyrosine 896)	P35568
JNK1	c-Jun N-terminal kinase 1	P45983
MEK	MAPK ERK kinase	Q02750
MEKK1	MAPK/ERK kinase kinase 1	Q13233

Continued on next page

Table S1 - continued from previous page

Abbreviation	Full name	UniProt ID
MK2	mitogen-activated protein kinase-activated protein kinase 2	P49137
MKK3/6	dual specificity mitogen-activated protein kinase kinase 3/6	P46734 / P52564
MKK4/7	dual specificity mitogen-activated protein kinase kinase 4/7	P45985 / O14733
NF-kB	nuclear factor kappa-B	P19838
p38	mitogen-activated protein kinase p38	Q16539
PDP1	par-domain protein 1	Q9TVY0
PER	period	P07663
PER/TIM-p	phosphorylated period-timeless complex	
RAF	Raf	P04049
RAS	Ras GTPase-activating protein	P01112
TGFα	transforming growth factor α	P01135
TNFα	tumor necrosis factor-α	P01375
TNFR	TNF receptor	P19438
TIM	timeless	P49021
VRI	vrille	O18660

Appendix D: Supplementary figures

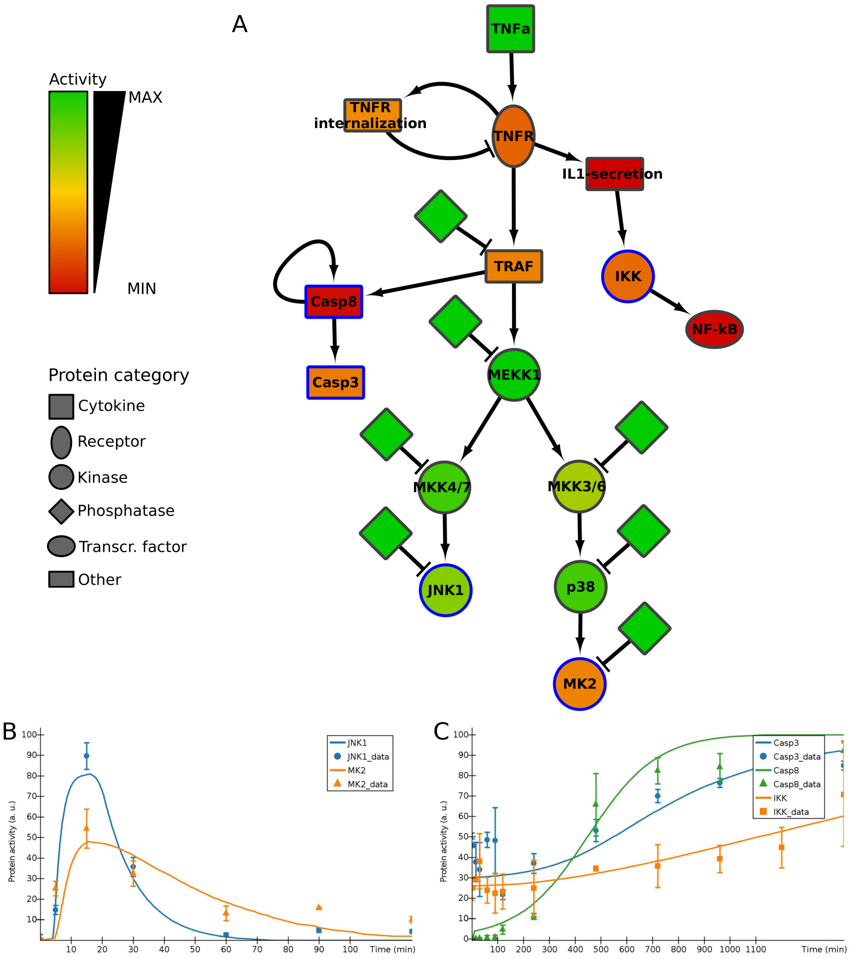

Figure S7: The model for the TNFα pathway in isolation. (A) Node colours represent the activity level of the corresponding modelled reactants at time $t = 10$ minutes after a stimulation of 100 ng/ml TNFα. **(B)** Early response: activity levels of JNK1 and MK2 in the first 2 hours of simulation. **(C)** Late response: activity levels of caspase-3, caspase-8 and IKK in the first 24 hours of simulation.

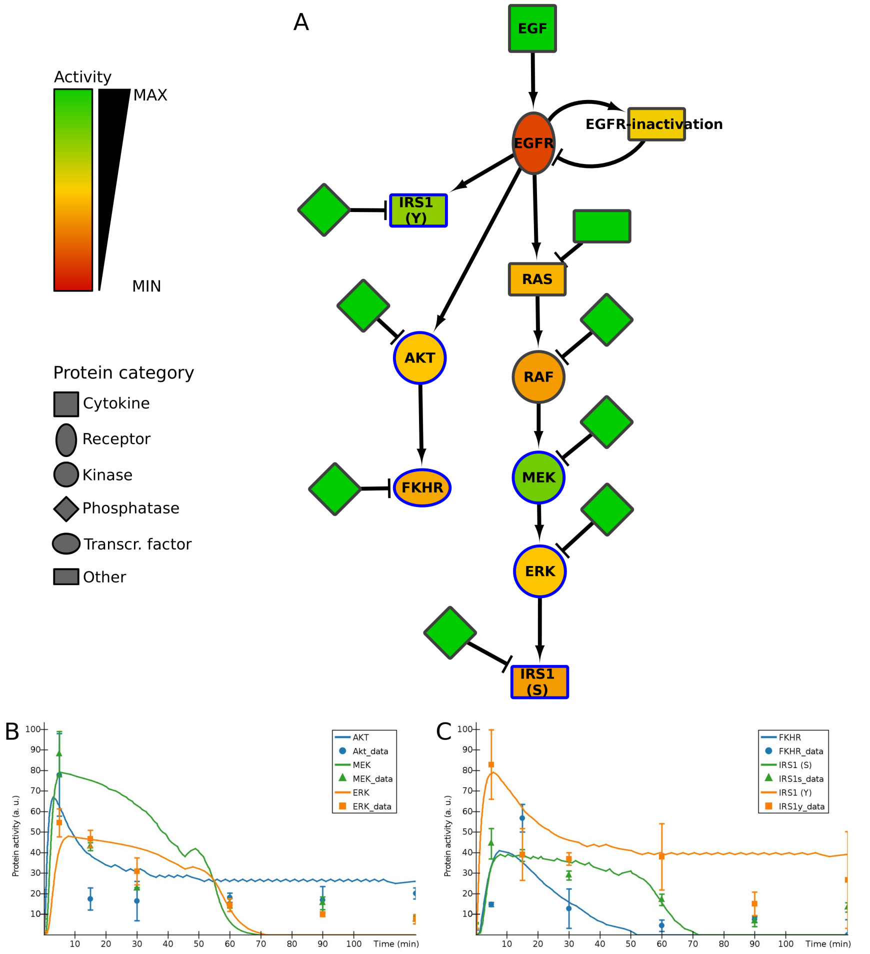

Figure S8: The model for the EGF pathway in isolation. (A) Node colours represent the activity level of the corresponding modelled reactants at time $t = 10$ minutes after a stimulation of 100 ng/ml EGF. **(B)** Activity levels of AKT, MEK and ERK in the first 2 hours of simulation. **(C)** Activity levels of FKHR, IRS1(S) and IRS1(Y) in the first 2 hours of simulation.

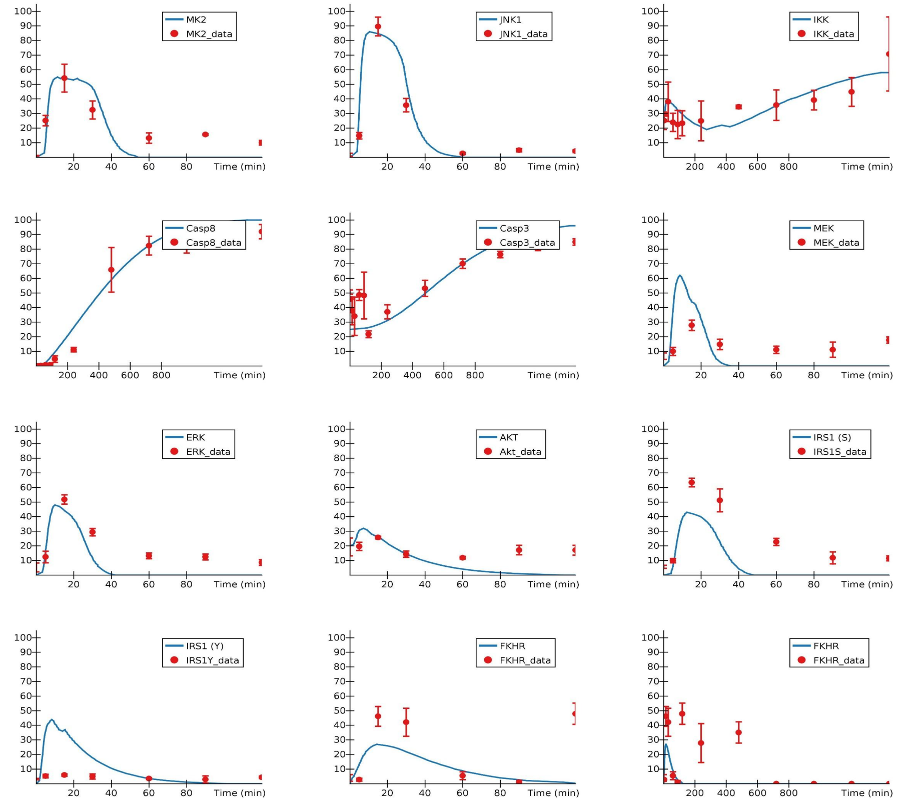

Figure S9: Comparison between the ANIMO model in Figure 5 and experimental data. Treatment condition: 100 ng/ml TNFα. In order to ease the comparison for earlier responses, the time span for those cases is less than 24 hours.

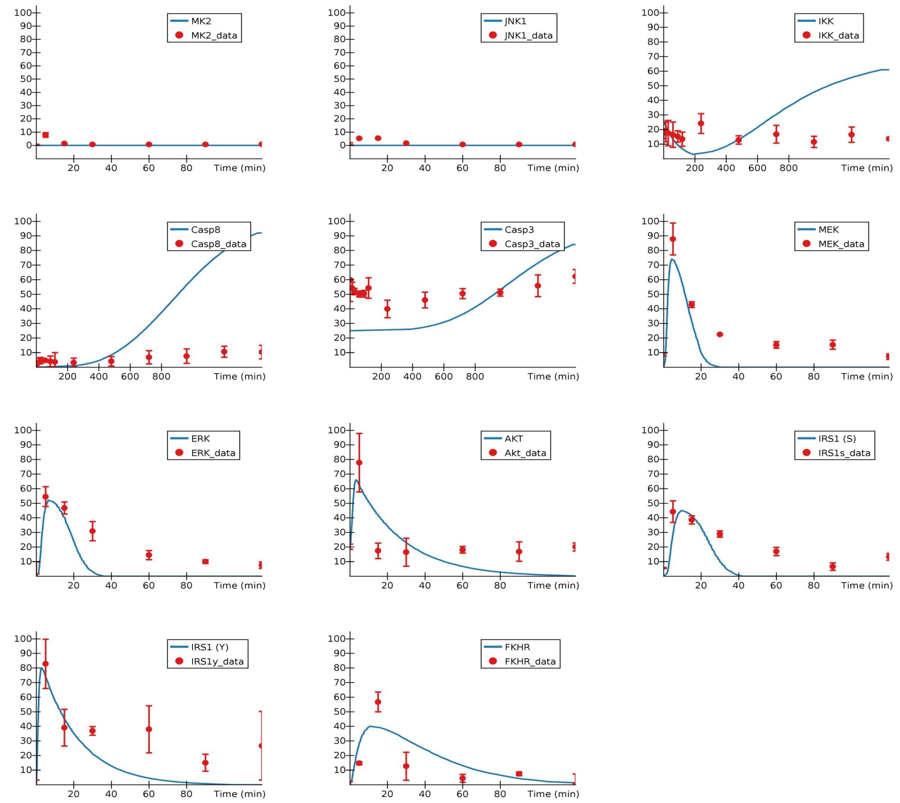

Figure S10: Comparison between the ANIMO model in Figure 5 and experimental data. Treatment condition: 100 ng/ml EGF. In order to ease the comparison for earlier responses, the time span for those cases is less than 24 hours.

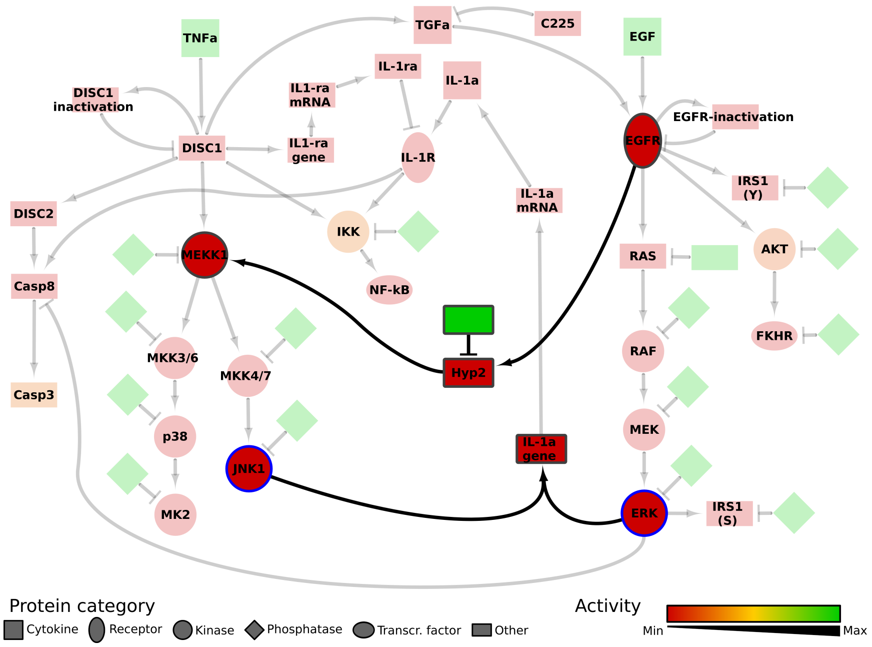

Figure S11: The merged model for the TNFα-EGF pathway with the two hypotheses highlighted. The first hypothesis is the dependence IL-1α expression on the combined activity of ERK and JNK1. The second hypothesis assumes an as yet unidentified protein (Hyp2) to link EGFR to MEKK1. Node colours represent initial activity levels.

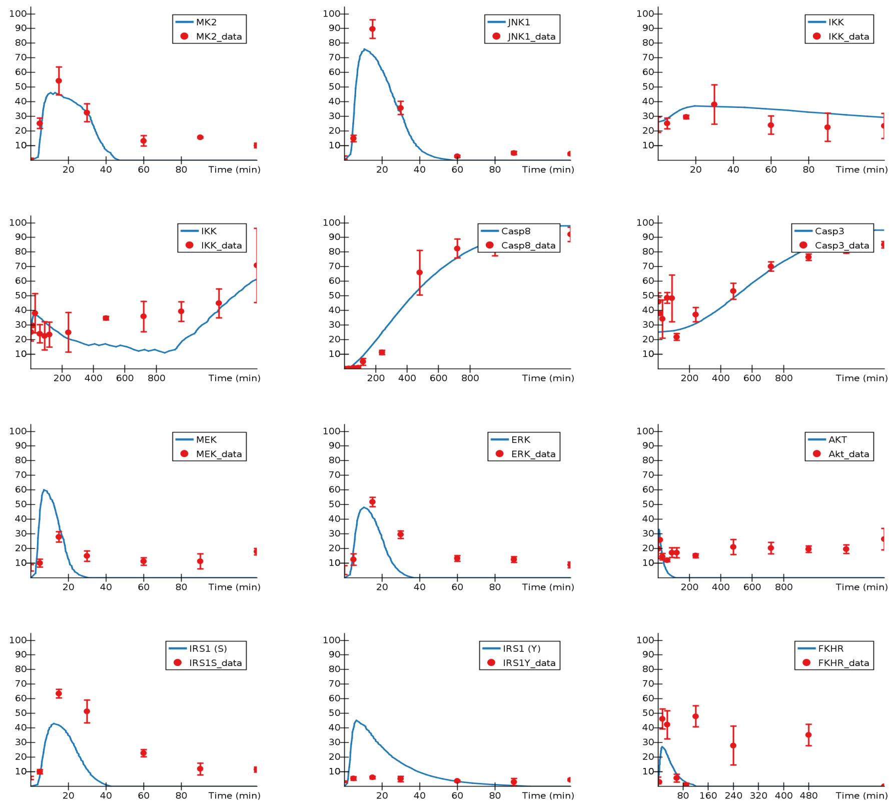

Figure S12: Comparison between the ANIMO model in Figure 6 and experimental data. Treatment condition: 100 ng/ml TNFα. In order to ease the comparison for earlier responses, the time span for those cases is less than 24 hours.

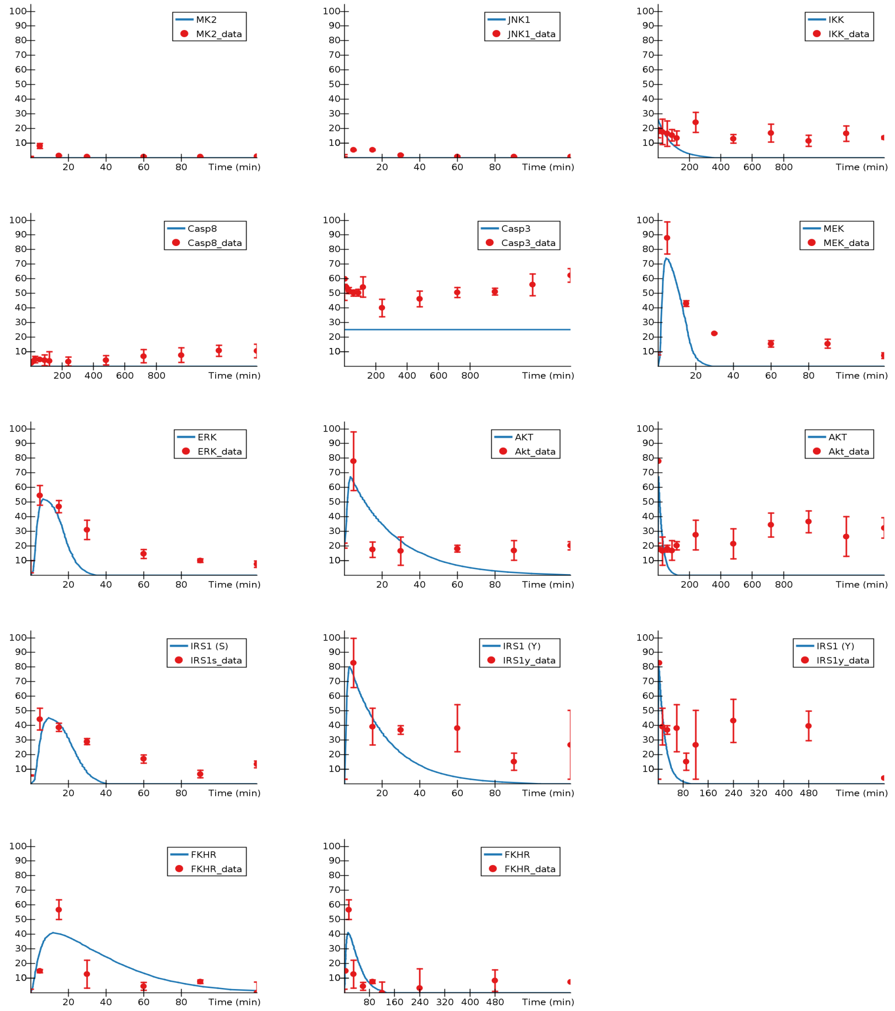

Figure S13: Comparison between the ANIMO model in Figure 6 and experimental data. Treatment condition: 100 ng/ml EGF. In order to ease the comparison for earlier responses, the time span for those cases is less than 24 hours.

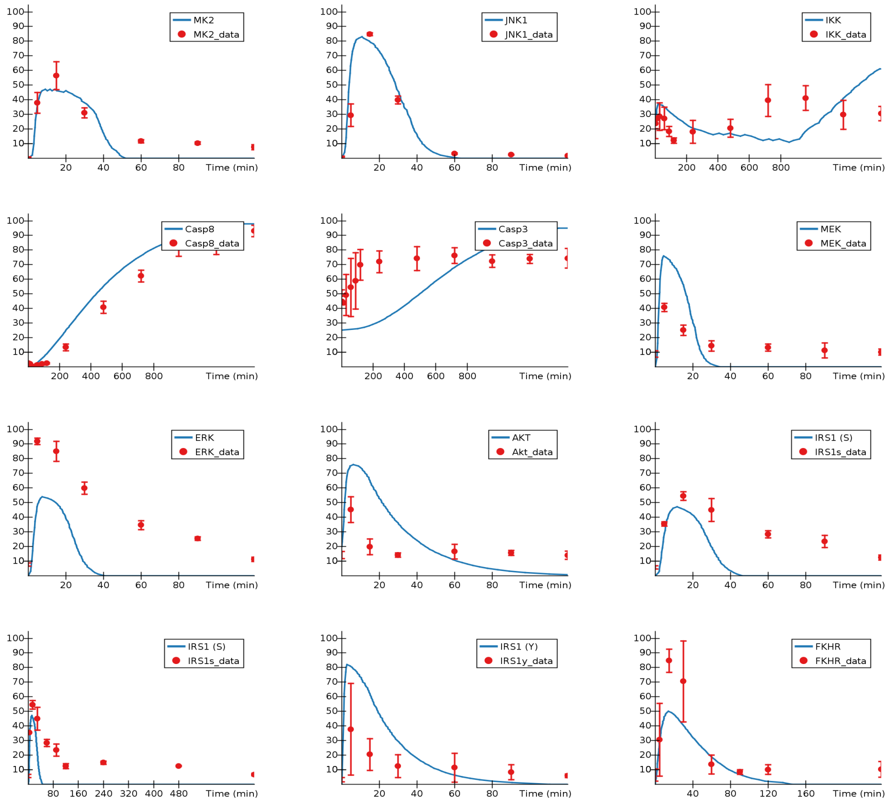

Figure S14: Comparison between the ANIMO model in Figure 6 and experimental data. Treatment condition: 100 ng/ml TNFα + 100 ng/ml EGF. In order to ease the comparison for earlier responses, the time span for those cases is less than 24 hours.
